# Decoding histone ubiquitylation

**DOI:** 10.3389/fcell.2022.968398

**Published:** 2022-08-29

**Authors:** Jennifer J. Chen, Dylan Stermer, Jason C. Tanny

**Affiliations:** Department of Pharmacology and Therapeutics, McGill University, Montreal, QC, Canada

**Keywords:** histone modification readers, ubiquitin signaling, histone ubiquitylation, PRC1 and PRC2 recruitment, DOT1l, 53BP1, BARD1 BRCT, Dnmt1

## Abstract

Histone ubiquitylation is a critical part of both active and repressed transcriptional states, and lies at the heart of DNA damage repair signaling. The histone residues targeted for ubiquitylation are often highly conserved through evolution, and extensive functional studies of the enzymes that catalyze the ubiquitylation and de-ubiquitylation of histones have revealed key roles linked to cell growth and division, development, and disease in model systems ranging from yeast to human cells. Nonetheless, the downstream consequences of these modifications have only recently begun to be appreciated on a molecular level. Here we review the structure and function of proteins that act as effectors or “readers” of histone ubiquitylation. We highlight lessons learned about how ubiquitin recognition lends specificity and function to intermolecular interactions in the context of transcription and DNA repair, as well as what this might mean for how we think about histone modifications more broadly.

## 1 Introduction

The genomic DNA of eukaryotes is organized into chromatin, a polymer whose fundamental structural unit is the nucleosome. The composition of the nucleosome core is essentially invariant across eukaryotes: an octamer composed of two copies of each of the core histone proteins H2A, H2B, H3, H4, and 146 base pairs of DNA wrapped around it. Linker DNA between neighboring nucleosomes varies in length from ∼10 to 50 base pairs depending on species and chromatin context and is bound by the linker histone H1 (Fyodorov et al., 2018). The packaging of genomic DNA into arrays of nucleosomes limits its access by cellular machineries that carry out transcription, replication, and repair (Kornberg and Lorch, 2020). Thus, mechanisms that allow access to DNA in the context of chromatin are integral to all of these processes. One mechanism that allows for dynamic organization of chromatin structure during these processes is histone post-translational modifications (PTMs). Histone PTMs are a key cellular mechanism for regulating chromatin structure and function. Generally, they are thought to comprise chromatin-based signaling networks in which modified histones form binding sites for chromosomal proteins that execute downstream functions (Strahl and Allis, 2000; Rothbart and Strahl, 2014). At the heart of these networks is the interface between modified histones and their cognate recognition proteins, often called histone modification “readers.” Many protein domains with dedicated reader functions have been identified, and numerous high-resolution structural views of reader-modification interactions are available. Reader domains that recognize distinct modifications have unique structural features. For example, reader domains for histone methylation can be distinguished based on the specific methylated site that they recognize: Plant homeodomain (PHD) fingers for histone H3 lysine 4 (K4) methylation, chromodomains for lysine 9 (K9) or lysine 27 (K27) methylation (sub-families of chromodomains are specific for each site), and proline-tryptophan-tryptophan-proline (PWWP) domains for lysine 36 (K36) methylation (Musselman et al., 2012). These domains allow histone modifications to guide biochemical activities on chromatin in multiple biological contexts.

In contrast, relatively few readers for histone ubiquitylation (Mattiroli and Penengo, 2021; Vaughan et al., 2021) have emerged from the detailed studies of this modification, and structural information illuminating ubiquitylated histone recognition has only recently become available, largely through cryo-EM studies (summarized in Table 1). Here we review the current state of knowledge of ubiquitylated histone readers, with a focus on their structure and function. This is a field that spans many aspects of chromatin biology and that illuminates novel functions for ubiquitin. Unlike other classes of histone modification readers, ubiquitylated histone readers do not share a particular domain or structural motif. As such, we discuss interactions between readers and ubiquitylated histones with a view toward identifying common themes and functional consequences.

**TABLE 1:** Summary of histone ubiquitylation sites and cognate reader proteins.

Modification	Reader	Experimental methods
H2AK119ub1	PRC2	Quantitative mass spectrometry (Kalb et al., 2014a), nucleosomal pulldown assays (Cooper et al., 2016), cryo-EM structure (Kasinath et al., 2021)
	PRC1	Immunofluorescence (Arrigoni et al., 2006), Nucleosomal pulldown assays (Zhao et al., 2020)
	RSF1	Quantitative mass spectrometry (Zhang Z. et al., 2017), pulldown assay (Zhang Z. et al., 2017)
	DNMT3A	ChIP-seq (Weinberg et al., 2021), dCypher nucleosome binding assay (Weinberg et al., 2021)
	ZRF1	Affinity purification (Richly et al., 2010), pulldown assays (Richly et al., 2010)
H2AK13/15ub1	53BP1	Pulldown assays (Fradet-Turcotte et al., 2013; Wilson et al., 2016), cryo-EM (Wilson et al., 2016), isothermal calorimetry (Wilson et al., 2016), bio-layer interferometry (Wilson et al., 2016)
	RNF169	Pulldown assays (Panier et al., 2012; Hu et al., 2017), isothermal calorimetry (Hu et al., 2017), NMR structure (Hu et al., 2017)
	RAD18	Pulldown assays (Hu et al., 2017), isothermal calorimetry (Hu et al., 2017), NMR structure (Hu et al., 2017)
	BARD1	Pulldown assays (Becker et al., 2021), electrophoretic mobility shift assays (Becker et al., 2021; Dai et al., 2021), isothermal calorimetry (Dai et al., 2021), microscale thermophoresis (Dai et al., 2021), cryo-EM structure (Dai et al., 2021)
H2AK127/129ub1	SMARCAD1	Pulldown assays (Densham et al., 2016)
	USP48	Cleavage assays (Uckelmann et al., 2018), gel-shift assays (Uckelmann et al., 2018), surface plasmon resonance (Uckelmann et al., 2018)
H2BK120ub1	Dot1L	Cryo-EM structures (Anderson et al., 2019; Jang et al., 2019; Valencia-Sánchez et al., 2019; Worden et al., 2019)
	COMPASS	*In vitro* histone methyltransferase assay (Hsu et al., 2019) (Kim et al., 2013), cryo-EM structure (Hsu et al., 2019; Worden et al., 2020)
	MLL complexes	Cryo-EM structure (Xue et al., 2019), *in vitro* histone methyltransferase assay (Xue et al., 2019)
	FACT	Has not yet been identified to bind directly to the ubiquitylated H2B
	SWI/SNF	Quantitative mass spectrometry (Shema-Yaacoby et al., 2013), co-immunoprecipitation (Shema-Yaacoby et al., 2013), ChIP-qPCR (Shema-Yaacoby et al., 2013), NGS of preferentially interacting nucleosome modifications (Mashtalir et al., 2021)
	Chd1	Has not yet been identified to bind directly to the ubiquitylated H2B
H3K18/23ub1	DNMT1	*In vitro* methylation assay (Ishiyama et al., 2017; Li et al., 2018), co-immunoprecipitation (Nishiyama et al., 2013), far-western blotting (Nishiyama et al., 2013), histone binding assays (Qin et al., 2015), pulldown assay (Ishiyama et al., 2017), isothermal calorimetry (Ishiyama et al., 2017), x-ray crystallography (Ishiyama et al., 2017)
H3K14ub1	Clr4/SUV39H1	*In vitro* ubiquitylation assay (Oya et al., 2019), pulldown assay (Oya et al., 2019), *in vitro* histone methyltransferase assay (Oya et al., 2019; Stirpe et al., 2021), hydrogen-deuterium exchange coupled to mass spectrometry (Stirpe et al., 2021), isothermal calorimetry (Stirpe et al., 2021)
H3K23/36/37ub1	Gcn5	Co-immunoprecipitation (Zhang X. et al., 2017), sequential purification (Zhang X. et al., 2017), pulldown assay (Zhang X. et al., 2017)

### 1.1 Overview of protein ubiquitylation

Ubiquitin is a 76 amino acid protein that can be post-translationally attached to other proteins. Ubiquitin attachment to a target protein (ubiquitylation; also referred to as ubiquitination) occurs in three enzymatic steps and results in an isopeptide bond linking the carboxy terminus of the ubiquitin polypeptide to the terminal amino group of a lysine side chain in the target (Komander and Rape, 2012). First, the E1 ubiquitin activating enzyme activates the ubiquitin carboxy terminus through ATP-dependent formation of a thioester linkage with an active site cysteine side chain. The activated ubiquitin is transferred to an E2 ubiquitin conjugating enzyme via another thioester bond. Transfer to substrate is facilitated by an E3 ubiquitin ligase, which forms a complex with the E2 and interacts directly with substrates (Komander and Rape, 2012). Ubiquitylation is dynamically controlled by deubiquitylation enzymes (DUBs). DUBs are a diverse family of proteases with specificity for the isopeptide bond. Their specificity for particular ubiquitylated substrates is determined by their protein interaction networks (Mevissen and Komander, 2017).

Protein ubiquitylation is most well known as the first step in the ubiquitin-proteasome system for protein degradation, the primary mechanism for programmed protein turnover and protein quality control in eukaryotic cells. Protein recognition by the proteasome requires polyubiquitylation, in which chains of ubiquitin are formed through isopeptide linkages at lysines within ubiquitin itself. All seven lysines in the ubiquitin polypeptide can in fact be targeted for chain formation, although the canonical type of chains recognized by the proteasome are linked at lysine 48. Non-proteolytic signaling functions of ubiquitin regulate protein activity and protein-protein interactions in a range of biological contexts. These can involve polyubiquitylation, particularly lysine 63-linked chains, but also frequently depend on monoubiquitylation of specific lysines in target proteins (Komander and Rape, 2012; Salas-Lloret and González-Prieto, 2022). Since histone ubiquitylation comprises mainly monoubiquitylation events, these will be the focus of our discussion.

Studies of monoubiquitylation signaling outside the realm of histones have defined recurrent ubiquitin receptor motifs. One group of motifs consists of bundles of alpha helices, or in some cases a single alpha helix. These are commonly found in proteins involved in endocytosis and include UBA (ubiquitin-associated), CUE (coupling of ubiquitin to ER degradation), UIM (ubiquitin-interacting motif), and MIU (motif interacting with ubiquitin) domains (Husnjak and Dikic, 2012). A second group has a zinc finger as the central structural element. This group is exemplified by the UBZ (ubiquitin-binding zinc finger) domain of DNA polymerase eta that recognizes monoubiquitylated PCNA to facilitate post-replication DNA repair; orthologous UBZ domains are found in other repair proteins as well (Husnjak and Dikic, 2012). Most characterized ubiquitin receptor motifs contact a hydrophobic patch on the ubiquitin surface surrounding Ile44. Study of histone modification readers has revealed additional examples of canonical ubiquitin interaction modes, and has also identified several novel types of ubiquitin-binding motifs that have important chromatin regulatory functions.

## 2 Histone H2A ubiquitylation

Monoubiquitylation occurs on several residues in the N- and C-terminal tails of histone H2A. Ubiquitylation on a C-terminal lysine corresponding to K119 in mammals was the first protein-ubiquitin conjugate to be characterized (Goldknopf and Busch, 1977). Identification of the cognate E2 and E3 enzymes as Polycomb group regulators linked this modification to transcriptional repression (Wang et al., 2004). More recent work has identified additional sites of monoubiquitylation on both the H2A N-terminal and C-terminal tails with important roles in coordinating the DNA damage response (Mattiroli et al., 2012; Kalb et al., 2014b) (Table 1).

### 2.1 H2AK119ub1 readers

The H2AK119-specific E3 ligase Ring1B is a member of the Polycomb family of transcriptional repressors. The Polycomb family proteins, first discovered in the fruit fly *D. melanogaster*, coordinate formation of facultative heterochromatin in metazoans (Blackledge and Klose, 2021). They are essential for the maintenance of gene expression programs associated with embryonic development and in adult tissues. As such, Polycomb regulators are among the most frequently mutated proteins in human cancers. The mechanism of Polycomb repression remains a topic of intense study, and is known to involve the formation of a repressive chromatin structure that is epigenetically inherited through cell division. Polycomb proteins reside in one of two types of Polycomb repressive complexes in cells, termed PRC1 and PRC2; Ring1 enzymes are core components of PRC1. PRC2 complexes also have histone-modifying activity conferred by the Ezh2 histone H3 lysine 27 methyltransferase. A key DUB for H2AK119ub1 is BAP1, a tumor suppressor that restrains Polycomb silencing genome-wide (Campagne et al., 2019; Fursova et al., 2021). The PRC1 complex mediates compaction of nucleosome arrays *in vitro* and formation of intranuclear Polycomb clusters *in vivo* (Francis et al., 2004; Grau et al., 2011; Isono et al., 2013). These functions correlate with liquid-liquid phase separation properties of PRC1 (Plys et al., 2019). Chromatin compaction by PRC1 *in vitro* is independent of any histone modifying activities and is insensitive to removal of histone tails, suggesting that Polycomb-associated histone modifications are important for targeting silencing to the appropriate genomic regions (Francis et al., 2004).

PRC1 and PRC2 are functionally linked on chromatin through self-reinforcing regulatory loops involving histone reader interactions (Blackledge and Klose, 2021). Recruitment of PRC1 by PRC2 activity is well understood and is mediated by a chromodomain protein (one of the CBX family proteins in mammals) that reads methylated H3K27 (H3K27me) and that is a component of canonical PRC1 complexes. However, the reverse relationship was only more recently described, and involves H2AK119ub1 reader proteins that associate with the PRC2 complex. Intriguingly, variant PRC1 complexes, which lack a chromodomain subunit, can still recognize and propagate H2AK119ub1, suggesting that these complexes can function through a PRC2-independent pathway to generate a silent state. H2AK119ub1 also exerts transcriptional regulatory functions through reader proteins that are entirely independent of the PRCs (Table 1).

#### 2.1.1 PRC2

A functional link between H2AK119ub1 and the PRC2 complex was first demonstrated by proteomic analysis that identified factors enriched on recombinant nucleosome arrays modified with H2AK119ub1 (Kalb et al., 2014a). PRC2 components were identified among the most robust interactors using extracts derived from fly embryos or mammalian ES cells, and the AEBP2 and Jarid2 subunits showed the highest enrichment. Moreover, a reconstituted PRC2 complex containing both AEBP2 and Jarid2 was shown to methylate H3K27 more efficiently on H2AK119ub1-modified nucleosome substrates than on unmodified ones. Subsequent studies uncovered direct interaction of Jarid2 with H2AK119ub1, confirming this factor as a direct reader of this modification (Cooper et al., 2016). A consensus UIM (ubiquitin interaction motif) in Jarid2 was shown to interact with the Ile44 hydrophobic patch of ubiquitin on H2A. Furthermore, mutation of UIM residues impaired the PRC2 recruitment function of Jarid2 in cells.

**FIGURE 1:**
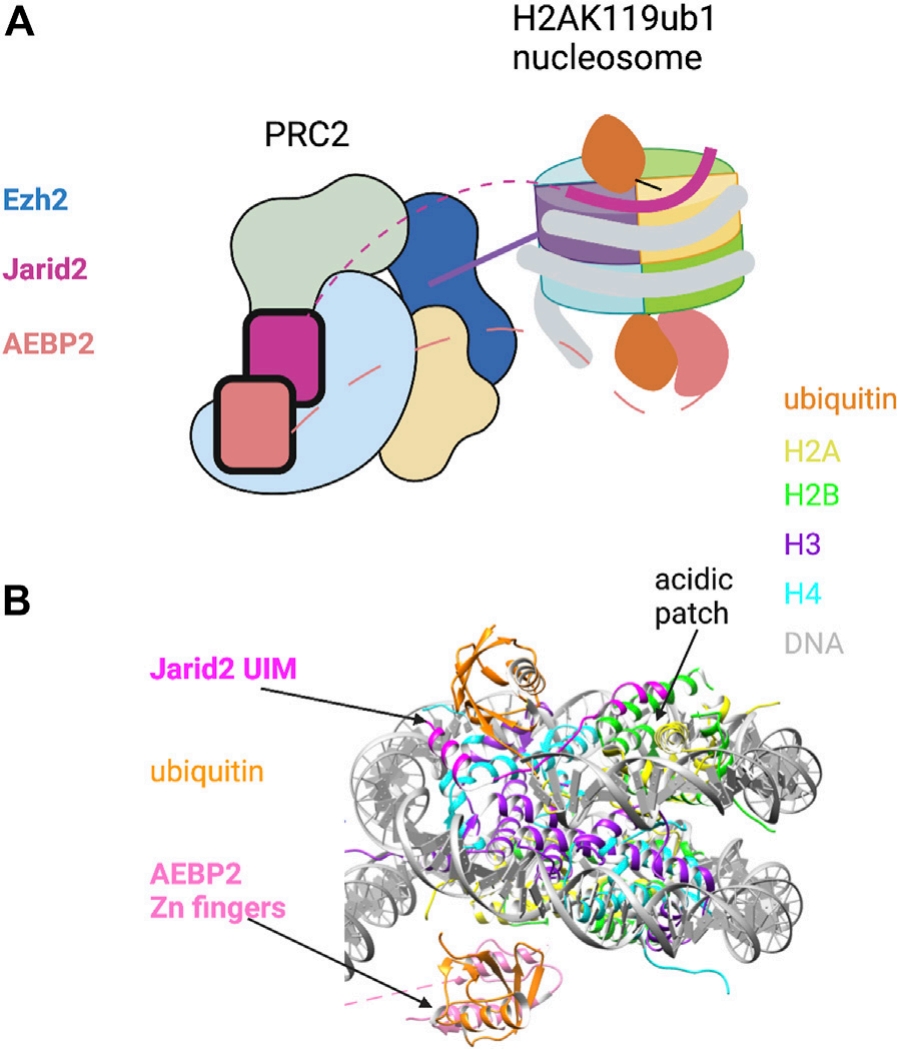
Recognition of H2AK119ub1 by the PRC2 complex. **(A)** Cartoon illustration of the cryo-EM structure of PRC2 bound to a H2AK119ub1 nucleosome (based on Kasinath et al., 2021). The H2AK119ub1 reader subunits Jarid2 and AEBP2 are highlighted; dashed lines denote presumed mobile segments of these proteins not visible in the structure. The Jarid2 UIM is depicted on the top surface by a thick line between ubiquitin and the acidic patch region. The AEBP2 zinc finger domain is shown on the bottom surface. The tail of histone H3 is shown positioned in the Ezh2 catalytic site. **(B)** Pymol rendering of the cryo-EM structure (PDB code 6WKR) showing the nucleosome and H2AK119ub1-binding modules. The H2A/H2B acidic patch on the nucleosome surface is also indicated. See text for details. Created with BioRender.com.

The cryo-EM-derived structure of PRC2 in complex with a H2AK119ub1-modified nucleosome offers a more complete picture of H2AK119ub1 recognition by PRC2 (Kasinath et al., 2021). The structure reveals direct contact between both Jarid2 and AEBP2 components with ubiquitin, suggesting a multi-valent H2Aub reader function for PRC2 (Figures 1A,B). Remarkably, both components engage H2AK119ub1 independently on opposite sides of the nucleosome, essentially forming a sandwich with the nucleosome in between them. On one side, the Jarid2 UIM forms an alpha helix that is wedged between the ubiquitin on H2A and nucleosomal DNA; this is stabilized by an adjacent Jarid2 segment bound to the “acidic patch” on the nucleosome surface (Figure 1B). The acidic patch is a cluster of acidic residues in H2A and H2B that forms a hotspot for factor engagement with the nucleosome (McGinty and Tan, 2021). On the opposite side of the nucleosome, two tandem zinc fingers in AEBP2 contact the ubiquitin attached to the other H2A subunit in the complex through the Ile44 hydrophobic patch. Additional contacts are also seen with H2A/H2B residues on this surface of the nucleosome. This structure explains the requirement for both Jarid2 and AEBP2 for PRC2 to respond to H2AK119ub1 *in vitro* and provides a general framework for understanding how PRC1 enzymatic activity regulates PRC2 in the context of Polycomb silencing.

Proteomic analysis of PRC2 has complicated this regulatory picture by demonstrating the existence of multiple PRC2 complexes composed of a group of shared core subunits (Ezh2, Suz12, Rbbp4, Rbbp7) and a set of auxiliary subunits. Jarid2 and AEBP2 are only present in one species of PRC2, termed PRC2.2, whereas PRC2.1 is endowed with Polycomb-like (PCL) subunits, DNA-binding proteins with preference for unmethylated CpG islands (Hauri et al., 2016). Thus, a regulatory role for H2AK119ub1 seems to only apply to a subset of PRC2 complexes *in vivo*, as confirmed by analysis of catalytic-dead Ring1B point mutants in mammalian ES cells (Blackledge et al., 2020; Tamburri et al., 2020). Interestingly, these point mutants also show complete loss of Polycomb silencing function even at PRC2.1 target genes, presumably because PRC1 functions are generally compromised (as is the case in a complete Ring1B knockout). This points to expanded roles for H2AK119ub1 beyond PRC2 activation.

#### 2.1.2 PRC1

As is the case for PRC2, PRC1 does not refer to a single entity but a family of related complexes (Blackledge and Klose, 2021). All PRC1 complexes contain Ring1B and one of six PCGF paralogs. In canonical PRC1 complexes, PCGF2 or PCGF4 are bound to a chromobox (CBX) subunit. CBX proteins are readers for methylated H3K27, and so canonical PRC1 complexes strongly depend on PRC2 enzymatic activity for their genomic localization and function. In contrast, variant PRC1 complexes contain PCGF1, 3, 5, or 6 bound to RYBP/YAF2. These complexes are independent of H3K27 methylation, and the H2AK119-specific ubiquitin ligase activity of these complexes renders overall levels of H2AK119ub1 essentially PRC2-independent in cells (Tavares et al., 2012; Blackledge et al., 2014). What drives recruitment of these complexes to chromatin if not PRC2? RYBP is a ubiquitin-binding protein: this interaction is mediated by its zinc-finger domain, which is similar in sequence to NZF zinc-fingers known to bind ubiquitin (Arrigoni et al., 2006). Ubiquitin binding by RYBP is important for PRC2-independent PRC1 recruitment in the context of X-chromosome inactivation in female ES cells (Almeida et al., 2017). Recently, RYBP was shown to interact directly with H2AK119ub1 nucleosomes *in vitro*; binding to H2Bub1 or unmodified nucleosomes in the same assays was comparatively weak. Moreover, RYBP was found to stimulate catalytic activity of PRC1 specifically in the presence of H2AK119ub1 nucleosomes (Zhao et al., 2020). *In vivo*, H2AK119ub1 and RYBP are interdependent for their chromatin association in ChIP-seq experiments. Taken altogether, these data support a model in which variant PRC1 propagates H2AK119ub1 through positive feedback. This model also emphasizes the central role H2AK119ub1 has in formation of Polycomb silencing assemblies in mammalian cells.

#### 2.1.3 Other H2AK119ub1 readers involved in transcriptional repression

The repressive function of H2AK119ub1 extends beyond its direct effects on PRC1 and PRC2. The ATP-dependent chromatin remodeling factor RSF (remodeling and spacing factor) was identified as a H2AK119ub1 reader in experiments that purified native chromatin highly enriched in H2AK119ub1 (Zhang Z. et al., 2017). RSF is a heterodimer consisting of a catalytic subunit (SNF2h) and a targeting subunit called RSF1; it is involved in multiple nuclear processes including transcription, DNA repair, and centromere function. RSF1 interacts specifically with H2AK119ub1 nucleosomes through its ubiquitylated-H2A-binding (UAB) domain. The UAB domain is highly conserved among RSF1 orthologs but lacks obvious sequence similarity to other ubiquitin-binding domains; structural analysis of this domain in complex with H2AK119ub1 would be revealing. ChIP-seq and RNA-seq analyses in cell lines demonstrated that RSF1 and Ring1B regulate overlapping cohorts of genes, suggesting that the RSF1-H2AK119ub1 interaction is physiologically relevant. *In vitro* transcription experiments on chromatin templates suggested that RSF directly represses transcription through H2AK119ub1. Further investigation is needed to determine how general the requirement for Ring1B or H2AK119ub1 is for RSF function, and, conversely, how important RSF might be for Ring1B functions in Polycomb silencing.

H2AK119ub1 was also recently shown to bridge the Polycomb system with *de novo* DNA methylation. DNA methylation at CpG dinucleotides is a key component of constitutive heterochromatin in mammals and is necessary for silencing of retrotransposons and repetitive elements (Edwards et al., 2017). Polycomb silencing generally operates independently of DNA methylation: variant PRC1 complexes directly bind to CpG islands at target gene promoters, but only when they are not methylated (Blackledge and Klose, 2021). However, the *de novo* DNA methyltransferase DNMT3A has a latent ability to target CpG islands occupied by H2AK119ub1 (Weinberg et al., 2021). This was revealed through analysis of mutant forms of DNMT3A in which the PWWP domain is impaired; such mutations are frequently found in patients with paragangliomas and microcephalic dwarfism (Weinberg et al., 2021). The DNMT3A PWWP domain is a reader for methylated H3K36, a histone mark that is depleted from promoter CpG islands. PWWP mutations cause DNMT3A redistribution and aberrant *de novo* DNA methylation at PRC1-regulated CpG island promoters. This effect is dependent on H2AK119ub1, as it is abolished by Ring1B removal, and is due to a direct interaction between DNMT3A and H2AK119ub1 nucleosomes that is mediated by a ubiquitin-dependent recruitment region (with no sequence homology to known ubiquitin-binding domains) in DNMT3A. Wild-type DNMT3A is preferentially localized by H3K36me through its PWWP domain. However, latent H2AK119ub1 recognition may be relevant in specific cell types or at certain times in development. For example, *de novo* methylation of CpG islands by DNMT3A has been reported in hematopoietic stem cells (Spencer et al., 2017). Additionally, during neuronal differentiation, many Polycomb-regulated gene promoters acquire DNA methyation (Mohn et al., 2008). Further studies are necessary to determine how specificity is conferred by the UDR of DNMT3A for H2AK119ub1, to what degree H2A monoubiquitylation contributes to DNMT3A localization in physiological settings, and how the balance between physiological and pathological targeting is achieved.

#### 2.1.4 ZRF1, a H2AK119ub1-reader interaction involved in transcriptional activation

Immunoaffinity purification of H2AK119ub1-containing chromatin identified zuotin-related factor 1 (ZRF1) (Richly et al., 2010). ZRF1 orthologs contain a DnaJ domain and two SANT domains; ubiquitin binding was mapped functionally to a different region of the protein that lacks sequence similarity to known ubiquitin-binding domains. ZRF1 occupies a subset of Ring1B/H2AK119ub1-occupied promoters, but seems to antagonize PRC1 binding to these targets, as suggested by a decrease in PRC1 occupancy upon ZRF1 overexpression. Moreover, upon differentiation of NT2 cells with retinoic acid, ZRF1 binding to its targets is enhanced, in concert with reversal of Polycomb-mediated repression and transcriptional activation. This suggests the intriguing model that H2AK119ub1 has a dual role in the Polycomb system, both in the establishment of the transcriptionally repressed state and in the activation of transcription at Polycomb-regulated genes following stimuli or differentiation. There are a number of outstanding mechanistic questions surrounding this model that have yet to be addressed. How does competition between ZRF1 and PRC1 operate? Can ZRF1 displace RYBP/YAF2 binding to H2AK119ub1 nucleosomes? How does this mechanism relate to H2AK119ub1 removal by the de-ubiquitylase BAP1, also proposed to be important for PRC1 antagonism during gene activation (Campagne et al., 2019)? As the derepression of Polycomb targets remains poorly understood, further insight into a role for H2AK119ub1 in this process would be of great interest.

### 2.2 H2Aub1 in DNA repair

Monoubiquitylation of H2A is a key component of the cellular response to DNA double strand breaks (DSBs) (Mattiroli and Penengo, 2021). These pathways do not directly involve H2AK119ub1 and instead lead to modification of distinct lysines on the N- and C-terminal H2A tails. Ubiquitylation of the N-terminal tail on lysine 13 or 15 (H2AK13ub1 or H2AK15ub1) occurs in the immediate vicinity of DSBs downstream of signaling cascades initiated by the damage checkpoint kinase ataxia telangiectasia mutated (ATM) and is catalyzed by the E3 ligase RNF168. (H2AK13ub1 and H2AK15ub1 are functionally interchangeable, so we will confine our discussion to the latter.) Readers for this modification regulate DSB repair pathway utilization and participate in both major DNA repair pathways in eukaryotic cells: non-homologous end joining (NHEJ) (53BP1), and homologous recombination (HR) (BARD1, RAD18, and RNF169). This suggests a general function for H2AK15ub1 in repair that is modulated by additional pathway-specific signals. These signals often work by resolving competition between 53BP1 and HR factors for binding to H2AK15ub1. They include the methylation state of the tail of histone H4, another chromatin feature sensed by repair factors that helps to dictate repair pathway choice. Ubiquitylation of H2A is also important for HR downstream of this decision point. BARD1, as part of a heterodimeric E3 ligase complex with BRCA1, catalyzes H2A C-terminal tail ubiquitylation at K125, K127 or K129, thereby promoting HR through the readers SMARCAD1 and USP48 (Table 1).

#### 2.2.1 53BP1

53BP1 engagement at chromatin surrounding a DSB is a major signal promoting NHEJ as it blocks resection at the broken ends, a necessary step for strand invasion leading to HR (Panier and Boulton, 2014). 53BP1 selectively binds to H2AK15ub1 and forms a scaffold, allowing other core NHEJ response proteins to assemble near the DSB end (Fradet-Turcotte et al., 2013). 53BP1 binding to the nucleosome involves recognition of two histone modifications: H2AK15ub1 and mono- or di-methylated H4K20 (H4K20me1/2) (Botuyan et al., 2006). The latter modification is not induced by DNA damage but is cell cycle regulated, such that high levels of methylation are present genome-wide only in cells in G1 (Saredi et al., 2016; Pellegrino et al., 2017). This helps to restrict 53BP1 binding and error-prone NHEJ from occurring when homologous chromosomes are present and HR is favorable.

**FIGURE 2:**
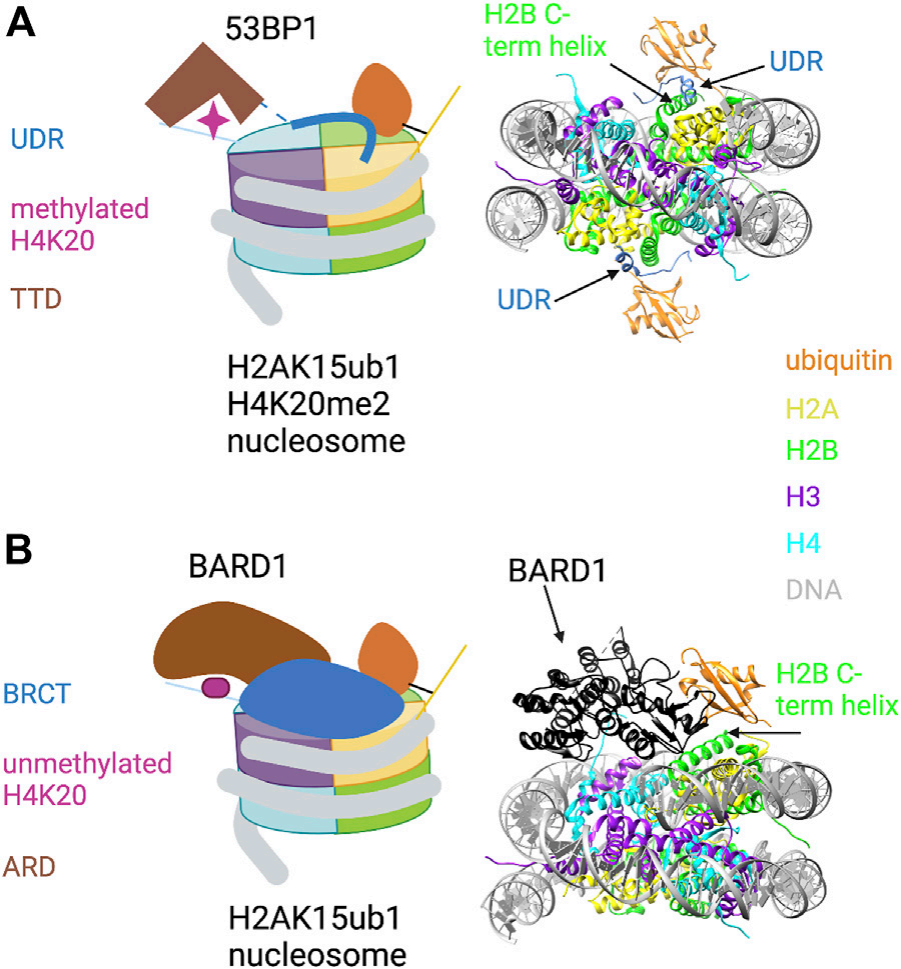
Key role of H2AK15ub1 in multi-valent nucleosome engagement by DNA repair factors. **(A)** Left: Cartoon illustration of the cryo-EM structure of the 53BP1 TTD-UDR region bound to a nucleosome harboring H2AK15ub1 and H4K20me2 (based on Wilson et al., 2016). H2AK15ub1 is shown connected to the H2A N-terminal tail and projecting over the nucleosome surface. The UDR is represented by a thick blue line contacting the H2B/H4 cleft, H2AK15ub1, and the acidic patch. The TTD bound to H4K20me2 is separated from the UDR by an unstructured region (dashed line). Right: Pymol rendering of the cryo-EM structure (PDB code 5KGF) showing the 53BP1 UDR bound to a H2AK15ub1 nucleosome. The TTD is not shown in this view. The H2B C-terminal helix is indicated. **(B)** Left: Cartoon illustration of the cryo-EM structure of the BARD1 ARD-BRCT region bound to a H2AK15ub1 nucleosome (based on Dai et al., 2021; Hu et al., 2021). Right: Pymol rendering of the cryo-EM structure (PDB code 7LYC) with the BARD1 ARD-BRCT region uniformly coloured black. The H2B C-terminal helix is indicated. See text for details. Created with BioRender.com.

53BP1 binds to H4K20me1/2 through its tandem Tudor domain (TTD) and to H2AK15ub1 through a ubiquitin dependent recruitment (UDR) motif located just C-terminal to the TTD (Wilson et al., 2016). The UDR was defined experimentally in domain swap experiments that conferred RNF168-dependent localization of a yeast 53BP1 ortholog to DNA damage foci in mammalian cells. Yeast lack RNF168 and H2AK15ub1, arguing that H2AK15ub1 affinity resides in this motif (Fradet-Turcotte et al., 2013). Although the UDR is not obviously similar to other ubiquitin-binding motifs, it is highly conserved among metazoan 53BP1 orthologs and is required for 53BP1 function in cells. Insight into how the 53BP1 UDR recognizes H2AK15ub1 came from a cryo-EM structure of a 53BP1 fragment containing the TTD and UDR bound to a nucleosome core particle modified with both H4K20me2 and H2AK15ub1 (Wilson et al., 2016) (Figure 2A). The ubiquitin is poorly resolved in the cryo-EM structure of the modified nucleosome alone, but association of the TTD-UDR fragment imparts structural rigidity, allowing clear inference of the ubiquitin conformation. The UDR forms an extended coil that is sandwiched between the nucleosome surface and the ubiquitin moiety, contacting both the ubiquitin Ile44 patch and a solvent-exposed cleft between histones H2B and H4. The C-terminus of the UDR forms a predicted alpha helix that contacts the H2A/H2B acidic patch. The structure also revealed roles for the nucleosome itself in positioning the H2AK15-linked ubiquitin. First, direct contacts were observed between ubiquitin and the H2B C-terminal alpha helix (Figure 2A, right). Second, ordering of the H2A N-terminal tail residues surrounding K15 conferred further structural rigidity and facilitated the UDR interaction; this involved interaction of the H2A R11 and R17 side chains with DNA. Thus, not only does the 53BP1 UDR engage in multivalent interactions with both ubiquitin and the nucleosome surface; it also potentiates ubiquitin interactions with other components of the nucleosome.

#### 2.2.2 RNF169 and RAD18

RNF169 is a paralog of RNF168, but its RING finger is not required for its function in DNA repair. Instead, DNA repair function requires a consensus MIU motif that is part of a ubiquitin-dependent recruitment module (UDM). The UDM directs RNF169 to double-strand breaks in a RNF168-dependent manner and binds specifically to H2AK15ub1 in a nucleosome context (Panier et al., 2012). Interestingly, RNF168 harbors a similar UDM, which presumably leads to amplification of the H2AK15ub1 signal at damaged sites. Unlike RNF169, RNF168 has a second UDM that is important for initial RNF168 recruitment to damaged chromatin through recognition of RNF8-dependent K63-linked polyubiquitylation on histone H1 (an event that is immediately downstream of the ATM kinase) (Doil et al., 2009; Stewart et al., 2009). RAD18 is similar to RNF169 in many respects: it is a E3 ubiquitin ligase harboring a UDM, its localization to DSBs is RNF168-dependent, and its positive role in HR requires ubiquitin binding but not E3 ligase activity (Huang et al., 2009; Panier et al., 2012).

The UDMs in RNF169 and RAD18 (and in RNF168) are bipartite in nature and are composed of a consensus ubiquitin-recognition motif and an adjacent nucleosome-binding motif termed the “LR” motif (LR refers to a conserved dipeptide within the motif). Both motifs are necessary for targeting to DSB foci in cells, and the LR motif can in fact be transferred to confer a similar localization on unrelated ubiquitin-binding proteins (Panier et al., 2012). The methyl-TROSY NMR structure of the RNF169 UDM bound to a H2AK15ub1 nucleosome elegantly validates this bipartite organization. The RNF169 MIU motif contacts the Ile44 patch of H2AK15-linked ubiquitin, and the interaction interface is oriented away from the nucleosome. The LR motif, extending from the MIU alpha helix, contacts the H2A/H2B acidic patch (Hu et al., 2017; Kitevski-LeBlanc et al., 2017). A similar division of labor applies to the RAD18 UDM. In this case, the ubiquitin-binding motif is a consensus UBZ motif; this contacts ubiquitin with the Ile44 patch facing the nucleosome, such that the UBZ helix is sandwiched between ubiquitin and the H2A/H2B acidic patch. The RAD18 LR motif then makes additional stabilizing contacts with the H2A/H2B acidic patch.

These structures, along with complementary biochemical experiments, suggest that RNF169 and RAD18 promote HR by competing with 53BP1 for binding to nucleosomes proximal to DSBs. There is striking overlap in binding sites for these factors on the nucleosome acidic patch. Moreover, RNF169 and RAD18 UDMs bind H2AK15ub1 nucleosomes (with or without H4K20me) with affinities that are two orders of magnitude greater than that of the 53BP1 TTD-UDR segment. Competitive binding assays *in vitro* showed that RNF169 or RAD18 displace 53BP1 from H2AK15ub1/H4K20me nucleosomes (Hu et al., 2017). This is consistent with experiments in which these factors displaced 53BP1 from DSB foci when overexpressed in cells (Poulsen et al., 2012; Helchowski et al., 2013; An et al., 2018; Nambiar et al., 2019).

#### 2.2.3 BARD1

Investigation of the recruitment of the BRCA1/BARD1 complex to sites of DNA damage revealed that the HR pathway utilizes a multivalent nucleosome recognition mechanism that parallels that of 53BP1. BRCA1 is a well-known tumour suppressor; its roles in promoting HR and opposing 53BP1 function have been studied extensively (Zhu et al., 2011; Witus et al., 2021). The BRCA1/BARD1 complex is a heterodimer of E3 RING-finger ubiquitin ligases; RING fingers in both proteins are required for ubiquitylation of its target sites on the C-terminus of histone H2A (see below). BARD1 is essential for the complex to bind to chromatin (Becker et al., 2021; Dai et al., 2021; Hu et al., 2021). Chromatin binding is conferred by the ankyrin repeat domain (ARD) and two tandem BRCA1 C-terminal domain (BRCT) repeats, two ubiquitous and versatile domains with a variety of interaction partners. In BARD1, the ARD binds to the unmethylated histone H4 tail (H4K20me0), and the BRCT domain binds to H2AK15ub1 (Nakamura et al., 2019; Becker et al., 2021). The interaction of the ARD with H4K20me0 stabilizes BRCA1/BARD1 at DSBs in the S and G2 phases of the cell cycle, times at which unmethylated H4K20 is abundant and HR is favored. This contrasts with the binding of 53BP1 to H4K20me in G1/M. A cryo-EM structure has been determined for the complex of the ARD-BRCT segment of BARD1 bound to a nucleosome ubiquitylated at H2AK15 and at the functionally related site H2AK13 (Dai et al., 2021; Hu et al., 2021) (Figure 2B). In the structure, the ARD makes extensive interactions with H4K20me0 by forming an acidic cavity around it; this may prevent methylation of H4K20. The ARD and the second (C-terminal) BRCT repeat are folded together in a V-shaped conformation that sits on the nucleosome surface; the BRCT interacts with the single ubiquitin moiety visible in the structure. This may correspond to H2AK13ub1 or H2AK15ub1, but binding seems to be restricted to one histone-linked ubiquitin. Interestingly, the structure reveals a unique ubiquitin-binding interface that is shared between the BRCT and the H2B C-terminal helix, with the Ile44 patch primarily contacted by the latter (Figure 2B, right). As in other H2AK15ub1 nucleosome structures, the H2A/H2B acidic patch is a critical interaction surface, making extensive contact with the second BRCT. BARD1 interaction with ubiquitin includes a direct contact with ubiquitin residue K63, implying that the complex would prevent K63-linked polyubiquitin chain formation. This may be relevant to how the BRCA1/BARD1 complex suppresses NHEJ in favor of HR, although further studies are needed to confirm this.

How is this mode of nucleosome recognition by BRCA1/BARD1 coupled to its enzymatic activity toward the H2A C-terminal tail? Remarkably, independent cryo-EM structures of the RING fingers of BRCA1 and BARD1 in complex with an unmodified nucleosome indicate binding to the same nucleosome surface as the ARD-BRCT regions (Hu et al., 2021). This may indicate that binding of the ARD-BRCT regions to one face of the nucleosome promotes binding of the RING fingers (and catalysis) on the opposite surface of the same nucleosome. An alternative model is that ARD-BRCT and the RING fingers create a bridge between neighboring nucleosomes, an intriguing possibility that remains to be investigated.

#### 2.2.4 SMARCAD1 and USP48

BRCA1/BARD1 is a E3 ubiquitin ligase that monoubiquitylates H2AK125/127/129 (Kalb et al., 2014b). The physiological relevance of this activity for BRCA1 function *in vivo* is controversial, although there is compelling evidence for some role for H2A monoubiquitylation downstream of BRCA1 in the context of DNA damage repair and heterochromatin formation (Zhu et al., 2011; Densham et al., 2016). There is also evidence pointing to two DNA repair factors as candidate readers for these H2A ubiquitylation sites. One is the SWI/SNF-related ATP-dependent chromatin remodeler SMARCAD1 (Densham et al., 2016). SMARCAD1 and BRCA1/BARD1 function in the same pathway in cells to regulate DNA resection at DSBs and promote HR. This seems to occur through nucleosome eviction and the redistribution of 53BP1 to sites distal from the DSB (Uckelmann et al., 2018). SMARCAD1 is an attractive reader candidate as it contains two ubiquitin-binding CUE domains and can bind to nucleosomes assembled *in vitro* with a H2A-ubiquitin fusion protein. Mutations in the CUE domains also compromise its function in cells (Densham et al., 2016).

A second repair factor that acts downstream of BRCA1/BARD1 activity is USP48, a DUB that removes H2AK125/127/129ub1 (Uckelmann et al., 2018). USP48’s recruitment to damage-proximal nucleosomes is markedly reduced in cells with depleted BRCA/BARD1. Importantly, the removal of H2AK125/127/9ub1 by USP48 prevents the recruitment of SMARCAD1 and subsequently, reduces chromatin remodeling around the damage site. 53BP1 also remains present around the site, which antagonizes DNA end resection and HR. In cells where either USP48 or 53BP1 are depleted, unregulated DNA end resection results in single-strand annealing, which is mutagenic for the cell. Interestingly, USP48 DUB activity toward K125/127/129ub1 requires the presence of an auxiliary ubiquitin somewhere on the nucleosome (i.e., the preferred substrate is a nucleosome that is multi-monoubiquitylated) (Uckelmann et al., 2018). The specificity of the auxiliary site has not been defined, but this suggests that USP48 may be a DUB for certain histone ubiquitylation sites and a reader for others. The requirement for an auxiliary ubiquitin may add an additional layer of regulation to the DNA repair response by allowing crosstalk between multiple ubiquitylated sites.

### 2.3 H2Aub1 summary

Detailed study of H2Aub1 readers has solidified the roles of these modifications as chromatin binding determinants, enhancing the affinity of a variety of factors (chiefly facultative heterochromatin proteins or DNA repair proteins) for specific genomic sites. For H2AK119ub1, a key issue moving forward is the extent to which readers outside of the Polycomb group of regulators contribute to its functions. Further illumination of the roles of the more recently discovered H2Aub1 modifications in the DNA damage response is also of great interest.

## 3 Histone H2B ubiquitylation

Histone H2B monoubiquitylation (H2Bub1) marks transcribed genes in all eukaryotes, suggesting that it is a fundamental feature of RNAPII transcription (Nickel et al., 1989; Tanny, 2014). The strong evolutionary conservation contrasts with ubiquitylation of H2A, which is absent in unicellular eukaryotes. The predominant form of H2Bub1 is modified on a conserved lysine corresponding to K120 in human H2B; this residue is in the H2B C-terminal helix, which, in the context of the nucleosome, is positioned on the nucleosome surface adjacent to the H2A/H2B acidic patch. H2Bub1 is catalyzed by the E2 ubiquitin-conjugating enzyme RAD6 and a dimeric E3 ligase complex composed of orthologs of yeast BRE1 (the RNF20/40 heterodimer in humans) (Robzyk et al., 2000; Hwang et al., 2003; Fuchs and Oren, 2014). These enzymes deposit H2Bub1 during the elongation phase of RNAPII transcription. The precise mechanisms that underlie this co-transcriptional process are still being elucidated, but it is clear that H2Bub1 is deposited through action of the core RNAPII transcription elongation machinery. The key molecular events are thought to be the following: phosphorylation of Spt5 by positive transcription elongation factor b (P-TEFb), binding of phosphorylated Spt5 by the elongation factor Rtf1, and stimulation of H2Bub1 catalysis through interactions between Rtf1, Rad6, and the H2A/H2B acidic patch (Tanny, 2014; Van Oss et al., 2016; Cucinotta et al., 2019). Polymerase Associated Factor (PAF) complex is important for stabilizing Rtf1 interaction with the elongating RNAPII (Mbogning et al., 2013; Cao et al., 2015; Van Oss et al., 2016). H2Bub1 is rapidly turned over during transcription and various DUBs have been implicated in this, most notably the DUB module of the SAGA co-activator complex (Morgan et al., 2016).

Although the functions of H2Bub1 during transcription are not fully understood, it clearly plays important gene regulatory roles. Ablation of H2Bub1 impairs embryonic development in the mouse and prevents stem cell differentiation (Fuchs et al., 2012; Karpiuk et al., 2012). Furthermore, altered H2Bub1 levels are associated with various cancers (Tarcic et al., 2016, 2017; Marsh and Dickson, 2019). Some of these effects are likely attributable to the direct link between H2Bub1 and histone methyltransferases specific for lysines 4 and 79 on histone H3 (H3K4 and K79 methylation) (Chandrasekharan et al., 2010). Like H2Bub1, these methylations are near-universal features of transcribed chromatin with key roles in embryonic development and cell growth (Shilatifard, 2012; Tanny, 2014; Krivtsov et al., 2017). H2Bub1 also acts independently of histone H3 methylation; relevant readers for these functions have not been identified (Tanny et al., 2007; Fleming et al., 2008; Minsky et al., 2008; Chandrasekharan et al., 2010). As there is significant evidence pointing to a role for H2Bub1 in regulating nucleosome structural transitions that accompany transcription, we highlight connections to ATP-dependent chromatin remodeling factors and chromatin assembly factors that may help to mediate these functions (Table 1).

### 3.1 Dot1L

The requirement of H2Bub1 for H3K4 and K79 methylation was the first example of regulatory crosstalk between modifications on different histone tails (Chandrasekharan et al., 2010). It was first established genetically in budding yeast; in this system, methyltransferase activity for H3K4 and H3K79 reside in one enzyme for each site (Set1 and Dot1, respectively). Subsequent work revealed a similar dependency in other organisms, although the presence of multiple H3K4 methyltransferases in metazoans complicates this picture. Recent biochemical experiments have demonstrated that H2Bub1 nucleosomes are the preferred substrate for Dot1 family H3K79 methyltransferases (comprising all known methyltransferases for this site), Set1 family H3K4 methyltransferases, and MLL family H3K4 methyltransferases. However, the magnitude of the stimulation conferred by H2Bub1 varies across different enzymes and experimental conditions (Wu et al., 2013; Xue et al., 2019; Kwon et al., 2020). Generally, MLL family methyltransferases exhibit smaller effect sizes than either Dot1 or Set1 enzymes.

H3K79 methylation is distributed in transcribed regions of genes in a pattern similar to that for H2Bub1 (Vlaming and van Leeuwen, 2016). H3K79 is located within the globular domain of histone H3 and is positioned on the nucleosome surface, suggesting physical proximity with H2Bub1 that would lend itself to H2Bub1-H3K79me crosstalk. Cryo-EM-derived structures show that the C-terminal portion of bound Dot1L (the human Dot1 ortholog) engages ubiquitin and the H2A/H2B acidic patch (Anderson et al., 2019; Jang et al., 2019; Valencia-Sánchez et al., 2019; Worden et al., 2019). Ubiquitin binding involves evolutionarily conserved helix and loop regions in Dot1L; these define a novel ubiquitin-binding motif that contacts a non-canonical hydrophobic patch on the ubiquitin moiety centered on Ile36. An invariant arginine adjacent to the ubiquitin-binding region is inserted into the acidic patch. Comparison with a structure of Dot1L bound to an unmodified nucleosome shows that the acidic patch interaction is maintained, and biochemical analyses have shown that Dot1L binds unmodified and H2Bub1 nucleosomes with similar affinities (McGinty et al., 2008; Valencia-Sánchez et al., 2019; Worden et al., 2019). However, bound Dot1L exhibits greater conformational flexibility on unmodified nucleosomes than on H2Bub1 nucleosomes, as indicated by cryo-EM and by site-specific crosslinking studies (Zhou et al., 2016; Valencia-Sánchez et al., 2019). H2Bub1 restricts this flexibility, allowing formation of a Dot1L-nucleosome complex that is compatible with activity. Interestingly, H2Bub1 is necessary, but not sufficient, for Dot1L activation. Transition to the active state also requires positioning of the Dot1L catalytic site through interaction with the N-terminal tail of histone H4, as well as a conformational change in histone H3 that positions the H3K79 side chain for catalysis. Although binding to H2Bub1 does not contribute to Dot1L affinity for the nucleosome, the binding energy derived from this interaction may pay for the conformational change that is necessary for activity (Worden et al., 2019).

### 3.2 COMPASS

**FIGURE 3:**
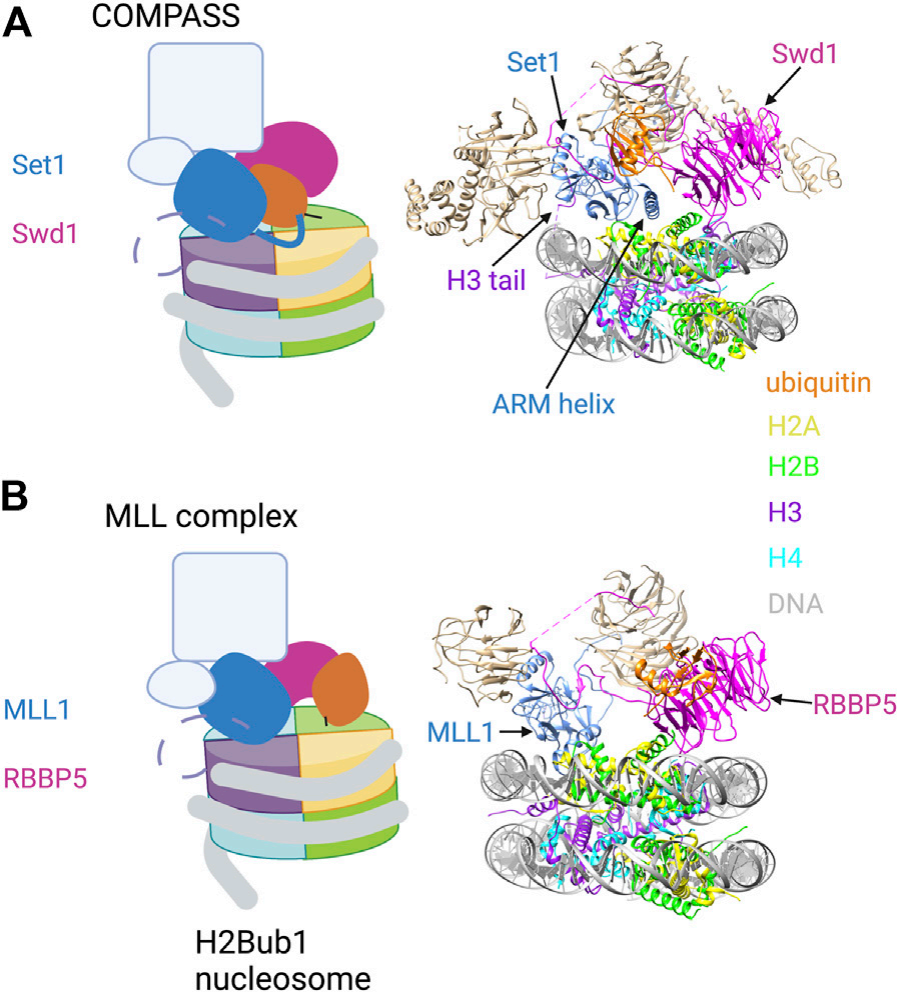
H2Bub1 recognition by COMPASS and MLL H3K4 methyltransferase complexes. **(A)** Left: Cartoon illustration of the cryo-EM structure of COMPASS bound to a H2Bub1 nucleosome (based on Hsu et al., 2019; Worden et al., 2020). The catalytic SET domain of Set1 and the Swd1 subunit are highlighted. The ARM helix is shown extending from the SET domain and contacting H2Bub1 and the acidic patch. The N-terminal tail of histone H3 is shown as a dashed line, with H3K4 engaged in the Set1 catalytic site. Right: Pymol rendering of the cryo-EM structure (PDB code 6VEN). Set1, Swd1, and ubiquitin components are coloured; additional auxiliary subunits are white. The ARM helix is indicated. **(B)** Left: Cartoon illustration of the cryo-EM structure of the MLL1 complex bound to a H2Bub1 nucleosome (based on Xue et al., 2019). The catalytic SET domain of MLL1 and the RBBP5 subunit are highlighted. The N-terminal tail of histone H3 is shown as a dashed line, with H3K4 engaged in the MLL1 catalytic site. Right: Pymol rendering of the cryo-EM structure (PDB code 6KIU). MLL1, RBBP5, and ubiquitin components are coloured; additional auxiliary subunits are white. See text for details. Created with BioRender.com.

H2Bub1 is also required for H3K4 di- and tri-methylation (H3K4me2/3), marks that are near-universal features of eukaryotic promoters. COMPASS family H3K4 methyltransferase complexes contain a catalytic subunit related to yeast Set1 and several auxiliary subunits, all of which are conserved from yeast to humans. Cryo-EM structures of yeast COMPASS bound to an H2Bub1 nucleosome were determined using a catalytically competent version of COMPASS which recapitulates H2Bub1 dependence *in vitro* (but lacking the N-terminal half of Set1 and two auxiliary subunits). COMPASS engages one surface of the nucleosome, with Set1 and Swd1 (ortholog of mammalian RBBP5) subunits making extensive contact with ubiquitin (Hsu et al., 2019; Worden et al., 2020) (Figure 3A). The tail of histone H3 loops between the gyres of the DNA superhelix to position H3K4 in the Set1 active site. Set1 interacts with ubiquitin via an alpha helix that includes the arginine-rich motif (ARM); this region of the protein is immediately adjacent to the catalytic SET domain and has been implicated in H2Bub1-dependent activity in biochemical assays (Kim et al., 2013). The hydrophobic C-terminal end of the ARM helix engages the Ile36 patch of ubiquitin, whereas the N-terminal portion of the helix extends over the nucleosome surface and makes electrostatic contact with the H2A/H2B acidic patch (Figure 3A, right). Swd1 is a key organizing component of COMPASS, making contacts with almost all the other subunits. In the nucleosome bound complex, the central ß-propeller domain of Swd1 interacts with all four core histones and with DNA, whereas the N- and C-terminal extensions contact the Ile44 hydrophobic patch of ubiquitin.

As is the case for Dot1L, H2Bub1 does not alter COMPASS binding affinity for nucleosomes, suggesting that H2Bub1 affects COMPASS catalytic activity (Worden et al., 2020). A comparison to the structure of COMPASS bound to an unmodified nucleosome reveals that although the overall structures are very similar, H2Bub1 induces folding of the C-terminal half of the ARM helix. This is associated with stabilization of the N-terminal portion of the SET domain and the H3 N-terminus in the active site, likely facilitating catalysis (Hsu et al., 2019).

### 3.3 MLL complexes

MLL complexes are unique to metazoans and are organized around an MLL H3K4 methyltransferase subunit, so named for the involvement of these factors in mixed lineage leukemia. Catalytic subunit aside, MLL complexes have a similar composition to COMPASS, including several shared auxiliary subunits (Krivtsov et al., 2017). These conserved subunits (WDR5, RBBP5, ASH2L, and DPY30), along with the MLL1 SET domain, constitute a fully active form of the MLL1 complex that was analyzed by cryo-EM. Structures of the complex bound to an H2Bub1 nucleosome have revealed a key role for RBBP5 in nucleosome and ubiquitin recognition (Figure 3B). This is similar to its role in COMPASS complexes, albeit with a distinct mode of ubiquitin binding (Xue et al., 2019). In the MLL1 structure, a helical insertion within the ß-propeller domain packs against the Ile44 hydrophobic patch of ubiquitin, an interaction that is stabilized by adjacent electrostatic contacts (Figure 3B, right). There is considerable plasticity in the RBBP5-H2Bub1 interaction, as alternate ß-propeller surfaces contact ubiquitin in some cryo-EM images.

The RBBP5 ß-propeller domain contacts the nucleosome surface in the H2Bub1 nucleosome complex at the H2B-H4 cleft, with secondary contacts proximal to H3K79 and the H2B C-terminal helix. The catalytic SET domain of the MLL1 subunit is also closely engaged with the nucleosome surface through contacts with the C-terminal helix of histone H2A, with H3K4 looping between the DNA gyres into the active site (as observed for COMPASS). In this structure, there is no close contact between the catalytic domain of the complex and H2Bub1 (Figure 3B, right). Interestingly, this catalytically engaged structural arrangement is also observed in some cryo-EM images of MLL1 complex bound to an unmodified nucleosome. However, complexes with an unmodified nucleosome also adopt an alternate conformation (not observed with H2Bub1 nucleosomes) in which RBBP5 and MLL1 SET domain are not in close contact with the nucleosome surface. In this conformation, RBBP5 is pushed toward the periphery of the nucleosome and primarily contacts DNA (Xue et al., 2019). Thus, these structures point to another example of H2Bub1 favoring an active conformation of an enzyme complex.

### 3.4 FACT

It is clear that H2Bub1 also functions independently of H3K4me and H3K79me to regulate gene expression and chromatin structure, but the relevant mechanisms remain uncertain. Connections between H2Bub1 and the Facilitates chromatin transcription (FACT) complex have been documented in several model systems. FACT is a histone chaperone complex composed of Spt16 and SSRP1/Pob3 subunits. Its primary function *in vivo* is to maintain nucleosome structure during RNAPII transcription and DNA replication (Formosa and Winston, 2020). *In vitro* and *in vivo* experiments have demonstrated that RNAPII elongation through chromatin is associated with removal and re-deposition of H2A/H2B dimers in transcribed nucleosomes (Belotserkovskaya et al., 2003; Ramachandran et al., 2017; Yaakov et al., 2021). Subnucleosomal particles in which one of the two H2A/H2B dimers is missing (called hexasomes) are intermediates of this exchange process and are enriched in transcribed genes. RNAPII elongation is preferentially associated with loss of the promoter-distal H2A/H2B dimer (Ramachandran et al., 2017). FACT is the primary histone chaperone implicated in ensuring this exchange occurs in a manner that preserves genic nucleosome structure (Ramachandran et al., 2017). Evidence linking H2Bub1 to FACT includes the following: H2Bub1 occupancy is highly correlated with that of elongating RNAPII *in vivo* (Fuchs et al., 2014); loss of H2Bub1 and FACT both result in disruption of chromatin structure in gene coding regions (Fleming et al., 2008; Chandrasekharan et al., 2009; Batta et al., 2011; Murawska et al., 2020); H2Bub1 stimulates FACT-dependent transcription through nucleosomes *in vitro* (Pavri et al., 2006); H2Bub1 influences FACT-dependent nucleosome assembly *in vitro* (Murawska et al., 2020). FACT can also stimulate deubiquitylation of H2Bub1 by the DUB Ubp10 (Nune et al., 2019). It remains unclear to what extent FACT acts as a bona fide H2Bub1 “reader.” The unstructured C-terminal domains of both Spt16 and SSRP1/Pob3 bind free H2A-H2B dimers in the context of a partially unfolded nucleosome, an interaction that is thought to represent a disassembly/reassembly intermediate (Kemble et al., 2015; Liu et al., 2020; Farnung et al., 2021). These interactions involve key DNA-binding residues on H2A and H2B, and likely shield the dimer from DNA interactions that could interfere with co-transcriptional nucleosome disassembly/reassembly (Liu et al., 2020). How H2Bub1 may directly impinge on these interactions is unclear and will require further structural analysis. *In vivo* studies in other systems suggest that the functional similarities of H2Bub1 and FACT could involve effects on other regulators (Sanso et al., 2012, 2020).

### 3.5 ATP-dependent chromatin remodelers

H2Bub1 has been implicated in the function of various ATP-dependent chromatin remodelers. A proteomic approach to isolate human proteins that bound preferentially to nucleosome arrays harboring H2Bub1 identified the SWI/SNF complex (Shema-Yaacoby et al., 2013). SWI/SNF and RNF20/40 were shown to jointly promote transcription of a subset of genes, but the mechanistic basis for this, and whether SWI/SNF interaction with the nucleosome is directly impacted by H2Bub1, have yet to be established. *In vitro*, H2Bub1 nucleosomes have been shown to be refractory to remodeling by several ATP-dependent remodelers, including ISWI and, interestingly, SWI/SNF (Dann et al., 2017; Mashtalir et al., 2021). Follow-up on these studies is needed to determine the *in vivo* significance of these effects.

The closest functional relatives to H2Bub1 among ATP-dependent remodelers are those related to budding yeast Chd1. Chd1 and related orthologs are involved in nucleosome organization within genes, and have regulatory links to H2Bub1 (Hennig et al., 2012; Lee et al., 2012; Pointner et al., 2012; Smolle et al., 2012; de Dieuleveult et al., 2016). Chd1 also physically interacts with FACT, and is required for FACT distribution along transcribed genes (Farnung et al., 2021; Jeronimo et al., 2021). *In vitro* assays have demonstrated that Chd1 remodeling activity is stimulated 2-3 fold by installation of H2Bub1 on at least one of the two H2A/H2B dimers in the nucleosome (Levendosky et al., 2016). This suggests that H2Bub1 may stimulate the nucleosome spacing and positioning function of Chd1 *in vivo*. However, cryo-EM structural analysis has shown that this effect is not due a H2Bub1 “reader” function of Chd1. Chd1 (trapped in a nucleotide-bound state using ADP-BeF) engages the nucleosome primarily through contacts with DNA, and unwraps two helical turns of DNA from one end. DNA unwrapping depends on bound nucleotide and is thus associated with the active state of the enzyme. The ATPase domain contacts the tail of histone H4 (as is observed for other remodelers) and helix 1 of histone H3, but there is no direct contact with H2A/H2B dimers or with ubiquitin (Sundaramoorthy et al., 2018). The basis for the effect of H2Bub1 on Chd1 activity remains unclear, but may be related to interactions between the ubiquitin and the unwrapped DNA. These interactions involve the K48 and R54 residues of ubiquitin and lead to repositioning of ubiquitin closer to the nucleosome periphery on the side on which DNA is unwrapped. Thus, H2Bub1 may enhance Chd1 activity by stabilizing the unwrapped state. This raises the possibility that H2Bub1 may affect chromatin remodeling activity independently of dedicated reader proteins, by influencing the intrinsic stability of nucleosome remodeling intermediates.

### 3.6 H2Bub1 summary

H2Bub1 has emerged as an allosteric regulator of multiple euchromatic histone methyltransferases. Its non-methyltransferase readers remain poorly defined. Further investigation of these mechanisms will likely lead to important insights into co-transcriptional regulation of gene expression.

## 4 Histone H3 ubiquitylation

Histone H3 ubiquitylation was first identified *in vivo* in elongating spermatids of rat testes (Chen et al., 1998), and its low abundance in somatic cells precluded detailed functional studies until recently. As is the case for H2Aub1, there is no single predominant site associated with H3 monoubiquitylation, and it is catalyzed by multiple E3 ligases. One key emerging function for H3ub1 is the regulation of heterochromatin formation. Whereas H2Aub1 regulates PRC-dependent facultative heterochromatin, H3ub1 has been implicated in constitutive heterochromatin that requires the DNA methyltransferase Dnmt1 and H3K9 methylation. This is exemplified in mammalian cells by the E3 ubiquitin ligase Uhrf1, which ubiquitylates lysines 14, 18 and 23 on H3 to promote recruitment of Dnmt1. H3K14ub1 also supports heterochromatin formation by activating H3K9 methyltransferases. In contrast, modification of H3K23, K36, and K37 by the E3 ubiquitin ligase Nedd4 may target the histone acetyltransferase GCN5 to stimulate gene expression (Table 1).

### 4.1 DNMT1

The DNA methyltransferase DNMT1 is a “maintenance” methyltransferase: its preferred substrate is unmethylated cytosine that is paired with a methylated cytosine on the opposite DNA strand. These “hemi-methylated” substrates arise during S phase of the cell cycle, immediately after replication of DNA regions containing methylated cytosines. This is consistent with the role of DNMT1 in maintaining DNA methylation through cell division and with its localization to replication foci in S phase (Leonhardt et al., 1992; Edwards et al., 2017).

Uhrf1 is the key DNMT1 regulatory factor mediating the maintenance methylation function of DNMT1 (Nishiyama et al., 2016). Uhrf1 is a multi-domain protein that includes a ubiquitin-like domain (UBL), a SET- and RING-associated (SRA) domain and an E3 RING ubiquitin ligase domain. Each of these domains promotes DNMT1 function through distinct but inter-related mechanisms. The UBL domain directly binds the replication foci targeting sequence (RFTS) domain of DNMT1. The SRA domain binds to hemi-methylated DNA; this binding, in conjunction with the RFTS interaction, restricts DNMT1 activity to hemi-methylated substrates and enhances its methylation maintenance function (Avvakumov et al., 2008; Li et al., 2018). Finally, the E3 RING domain activates and recruits DNMT1 by ubiquitylating histone H3, expanding the catalog of enzyme regulatory functions for histone ubiquitylation (Nishiyama et al., 2013; Qin et al., 2015).

**FIGURE 4:**
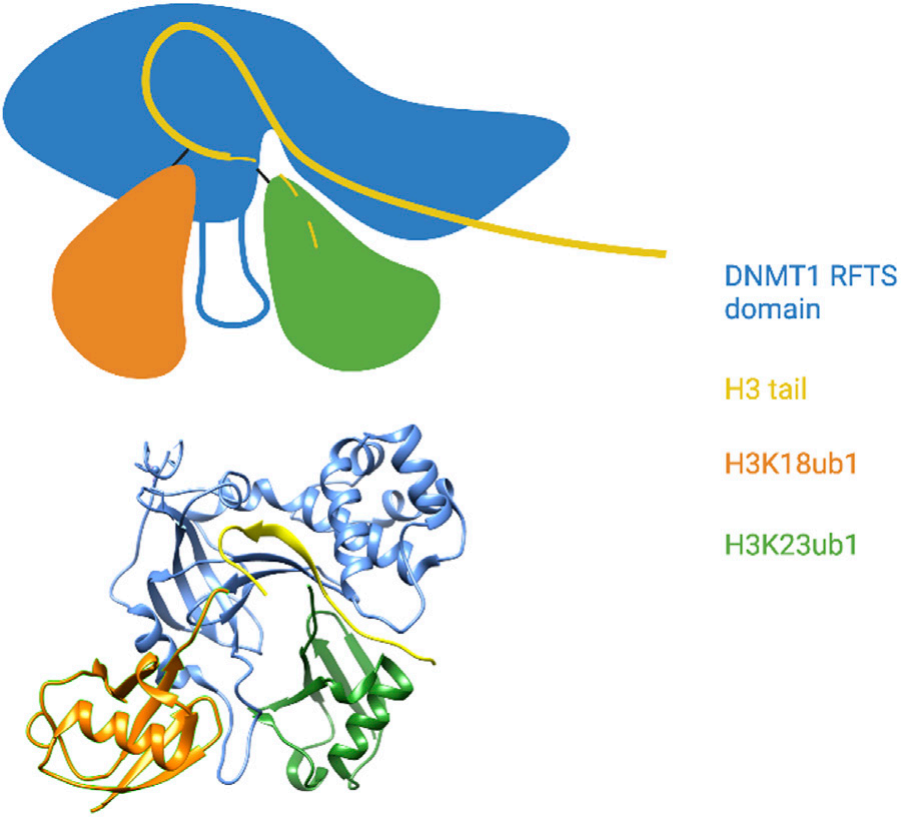
The DNMT1 RFTS domain reads a multi-monoubiquitylated histone H3 tail. Top: Cartoon illustration of the X-ray crystal structure of the DNMT1 RFTS domain bound to H3K18/K23ub1 (based on Ishiyama et al., 2017). The ubiquitin-binding URL is depicted projecting from the RFTS between the two ubiquitin moieties. The C-terminal end of the H3 tail was not visible in the structure and is shown as a dashed line. Close contact between the RFTS, the H3 tail, and H3K23ub1 is shown. Bottom: Pymol rendering of the X-ray crystal structure (PDB code 5WVO). See text for details. Created with BioRender.com.

Uhrf1 catalyzes monoubiquitylation of histone H3 on lysines 14, 18, and 23; appearance of these modified forms are dependent on ongoing DNA replication in a cell-free system derived from Xenopus oocytes (Nishiyama et al., 2013). Dnmt1 is the reader for these modifications, and specific binding of Dnmt1 to ubiquitylated H3 occurs through a consensus UIM found within the RFTS domain (Nishiyama et al., 2013; Qin et al., 2015). The preferred binding target of this UIM is a doubly monoubiquitylated form of the histone H3 tail, indicating that it engages two ubiquitin moieties simultaneously (Ishiyama et al., 2017). X-ray crystal structures of the human Dnmt1 RFTS domain bound to the histone H3 tail monoubiquitylated at lysines 18 and 23 reveals that the UIM takes the form of an extended loop (the ubiquitin recognition loop or URL) sandwiched between the two ubiquitins (Ishiyama et al., 2017; Li et al., 2018) (Figure 4). Hydrophobic residues on one side of the URL contact both the Ile44 patch and the Ile36 patch of H3K18ub1. The opposite side of the URL contacts an atypical interaction surface on H3K23ub1 consisting of Lys48 and Gln49, whereas the Ile44 patch of this ubiquitin is contacted by RFTS hydrophobic residues adjacent to the UIM. Interestingly, the RFTS domain also interacts with other parts of the H3 tail, as does H3K23ub1. The H3 tail is well-resolved in the structure and adopts a kinked conformation with a sharp (>90°) turn at Gly12 and Gly13. H3 residues N-terminal to this wind through a cleft between the RFTS (a region C-terminal to the URL) and H3K23ub1 (Ishiyama et al., 2017) (Figure 4). Thus, the DNMT1 ubiquitylated histone reader interaction involves a network of contacts between the RFTS domain, two adjacent ubiquitin moieties, and the histone H3 tail.

RFTS binding to H3ub not only targets Dnmt1 to S-phase chromatin, but also alleviates autoinhibitory binding of RFTS to the Dnmt1 catalytic pocket (Li et al., 2018). An independent interaction of RFTS with the UBL domain of Uhrf1 has a similar dual role, arguing that Uhrf1 employs multiple mechanisms to ensure Dnmt1 is specifically active at sites of DNA replication.

### 4.2 H3K9 methyltransferases

Uhrf1 also restricts Dnmt1 action to heterochromatic regions of the genome. This occurs through interaction between the Uhrf1 tandem Tudor domain (TTD) and methylated H3K9 (H3K9me), the major histone modification associated with constitutive heterochromatin (Rothbart et al., 2012, 2013).

Proteins related to Heterochromatin protein 1 (HP1) bind to H3K9me through their chromodomains and create a condensed chromatin structure through a mechanism that involves liquid-liquid phase separation (Larson et al., 2017; Strom et al., 2017; Sanulli et al., 2019). In mammalian cells, there are 5 different SET-domain containing methyltransferases that deposit H3K9me (SETDB1, SUV39H1, SUV39H2, GLP, and G9a). However, in fission yeast, there is only a single H3K9 methyltransferase, Clr4. Interestingly, Clr4 is found in a complex (the Clr4 methyltransferase complex or CLRC) that also contains a Cullin-RING ubiquitin ligase, the substrate of which was recently identified as lysine 14 of histone H3 (Oya et al., 2019).

H3K14ub1 greatly stimulates the methyltransferase activity of Clr4 toward H3K9 on the same histone tail *in vitro*, and Clr4 preferentially methylates H3K14ub1-containing nucleosomes (Oya et al., 2019; Stirpe et al., 2021). Biochemical studies indicate that a region of Clr4 adjacent to the SET domain (termed the ubiquitin-binding region or UBR), as well as a region distant from the catalytic domain near the N-terminus, are both involved in ubiquitin recognition; clarification awaits more comprehensive structural studies. Notably, a similar effect of H3K14ub1 is apparent on the activity of the mammalian ortholog of Clr4, SUV39H1 (at least *in vitro*), suggesting that ubiquitin regulation of H3K9me is a conserved feature of heterochromatin formation (Stirpe et al., 2021).

There are a number of outstanding questions that need to be resolved to fully appreciate the significance of this mechanism. *In vivo* studies in fission yeast show that H3K14ub1 is important at some, but not all, sites of heterochromatin, and leave open the possibility that Clr4 could be regulated by other ubiquitylated substrates (Oya et al., 2019). The effect of H3K14ub1 is selective *in vitro* as well: SUV39H1, which has a UBR that is homologous to that in Clr4, is stimulated by H3K14ub1, but G9a, which lacks this region, is not (Stirpe et al., 2021). It is also unclear as of yet what the identity of the relevant H3K14 ubiquitin ligase is in mammalian cells (although Uhrf1 would seem a promising candidate).

### 4.3 Gcn5

Nedd4-catalyzed H3 ubiquitylation has been associated with promoting transcriptional activation (Zhang X. et al., 2017). Upon glucose stimulation, Nedd4 catalyzes monoubiquitylation at multiple sites on H3 including lysines 23, 36, and 37. Loss of this activity is associated with a decreased expression of glucose-regulated genes and decreased levels of H3 acetylation, specifically at lysines 9 and 14 (K9/K14). This was shown to reduce tumour formation in cellular models. Crosstalk between H3 ubiquitylation and H3 acetylation was specifically linked to the histone acetyltransferase (HAT) Gcn5, which is known to preferentially acetylate H3K9/K14. When mutated, the loss of Gcn5 phenocopies the loss of Nedd4 and H3 ubiquitylation. Co-immunoprecipitation and *in vitro* assays showed that Gcn5 preferentially interacts with monoubiquitylated H3. The mechanistic basis for H3ub-dependent stimulation of Gcn5 has yet to be determined and awaits more detailed biochemical analyses.

### 4.4 H3ub1 summary

By virtue of its interaction with Dnmt1, H3ub1 has emerged as an important regulator of constitutive heterochromatin formation in metazoans. Further investigation of H3ub1 regulation of histone H3 lysine 9 methyltransferases may reveal an even broader heterochromatin function. It will also be of interest to determine the extent to which other H3ub1 modifications could have roles in gene activation.

## 5 General conclusion and perspectives

We have highlighted diverse modes of ubiquitylated histone recognition by structurally distinct reader proteins. Three common themes have emerged from the in-depth biochemical and structural analyses that will be useful in guiding future studies of this important class of regulators.

### Ubiquitylated histone readers also recognize other sites on the nucleosome

Binding specificity of histone methylation or acetylation readers is dictated by the presence of the modification and the amino acids surrounding the modified site. However, the affinity of reader domains for isolated histone tails is often weak: K_d_ values for these interactions commonly reach the millimolar range. Investigation of reader interactions with modified histones in the context of the nucleosome have demonstrated that several reader proteins interact with nucleosomal DNA cooperatively with the modified histone tail, suggesting that singular interaction of a reader with a modified histone is not sufficient to drive physiological interactions on chromatin (Musselman et al., 2014; Morrison et al., 2017). Readers of histone ubiquitylation exhibit similar cooperativity, engaging ubiquitin together with the nucleosome while exhibiting little affinity for free ubiquitin. The most common cooperative interaction of this type involves ubiquitylation of histone H2A or H2B and the H2A/H2B acidic patch on the nucleosome surface. This has been most clearly defined for readers of H2AK15ub1, which have a UDM consisting of separable but linked motifs recognizing either ubiquitin or the acidic patch (53BP1, RNF169, RAD18) (Panier et al., 2012; Mattiroli and Penengo, 2021). The acidic patch also serves as a critical partner in ubiquitylated histone recognition by a more loosely associated group of readers, each with unique structural determinants for ubiquitin and nucleosome recognition. These include H2Aub1 readers BARD1 and Jarid2, as well as H2Bub1 readers Dot1L and Set1 (Cooper et al., 2016; Worden and Wolberger, 2019; Hu et al., 2021). The strong association between H2Aub1/H2Bub1 recognition and the acidic patch suggests that these modifications may have more general roles in modulating the function of factors that engage this feature of the nucleosome surface. Expanded investigation of such factors, which continue to be identified through biochemical and proteomic studies, could point to new readers for histone ubiquitylation (Skrajna et al., 2020).

Studies thus far indicate that H3ub1 recognition only involves the H3 tail. The RFTS domain of Dnmt1 binds to free ubiquitin, as well as to the ubiquitylated H3 tail, consistent with the idea that it relies on binding determinants centered on the ubiquitylated site itself (Qin et al., 2015; Ishiyama et al., 2017). Ubiquitin also influences the activity of Clr4 in the context of the isolated H3 tail (Stirpe et al., 2021). It remains to be seen whether further study of these readers in a nucleosome context will capture additional important interactions.

### Histones and DNA can participate in ubiquitin recognition

An intriguing feature of some of the complexes with ubiquitylated histones is that the ubiquitin moiety itself contacts histones or nucleosomal DNA. H2AK15-linked ubiquitin contacts the H2B C-terminal helix when bound to the 53BP1 UDR or BARD1 BRCT domains (Wilson et al., 2016). H2B-linked ubiquitin contacts DNA unwrapped from the nucleosome by Chd1, whereas H3K23ub1 interacts with the H3 N-terminal tail when bound to the DNMT1 RFTS domain (Ishiyama et al., 2017; Sundaramoorthy et al., 2018). These interactions result from the specific biochemical context of the relevant complexes, but hint at the possibility that, under certain circumstances, ubiquitylated histones can alter chromatin structure on their own, independently of downstream readers. There is support for this idea from studies examining the effect of H2Bub1 on the biophysical properties of nucleosomes and nucleosome arrays *in vitro*. H2Bub1 decompacts nucleosome arrays, as assessed by analytical ultracentrifugation analysis; this effect was not observed with Hub1, a ubiquitin-like modifier protein that is not conjugated to histones in cells (Fierz et al., 2011). Decompaction is mediated by an acidic surface on ubiquitin (Glu16 and Glu18) that drives ubiquitin-ubiquitin electrostatic interaction (Debelouchina et al., 2017). Moreover, the intrinsic stability of nucleosomes has been shown to be enhanced by H2AK119ub1 and reduced by H2Bub1, although conflicting data from various studies preclude drawing a definitive conclusion (Fierz et al., 2012; Krajewski et al., 2018; Xiao et al., 2020). Nonetheless, these results demonstrate that direct interaction of ubiquitin with chromatin has potential functional consequences. Going forward, it will be important to evaluate more thoroughly their significance *in vivo*.

### Biochemical outcomes of reader interactions are different for different ubiquitylated histones

H2Aub1 enhances the affinity of its cognate reader proteins for the nucleosome. This has been studied extensively for readers of H2AK15ub1, establishing this modification and its readers as focal points in bringing mediators of the DNA damage response to sites of damage *in vivo* (Mattiroli and Penengo, 2021). Reader interactions with H2AK119ub1 are less well defined, but the evidence clearly points to enhanced affinity of Jarid2 for the nucleosome as important for the biological effects of H2AK119ub1 (Kalb et al., 2014a; Cooper et al., 2016). From this perspective, H2Aub1 readers align with previously defined readers of H3 and H4 tail modifications such as acetylation and methylation, which serve as binding sites for discrete protein modules. H3ub1 enhances affinity of the interaction of the DNMT1 RFTS domain with the H3 tail, suggesting that H3ub1 readers may be similarly classified (Ishiyama et al., 2017). In contrast, H2Bub1 does not enhance the affinity of interaction of COMPASS, MLL complexes, or Dot1L with the nucleosome, and instead acts allosterically, stabilizing active enzyme conformations (Worden and Wolberger, 2019). This sets H2Bub1 readers apart in a unique class, not only among readers of ubiquitylated histones but among histone modification readers in general. The notion that histone modifications act as allosteric modulators of reader proteins suggests that approaches aimed at identification and characterization of reader proteins need to encompass nuanced, in-depth analyses that go beyond interaction affinity.

Histone ubiquitylation has a clear connection to human disease, as demonstrated by the gain of H2Aub1 and loss of H2Bub1 in various malignancies (Marsh and Dickson, 2019). As reader interactions with histone modifications emerge as druggable targets in human disease (Arrowsmith and Schapira, 2019), further study of histone ubiquitylation readers and their regulatory mechanisms is likely to result in clinically translatable insights.

## References

[B1] Almeida M. Pintacuda G. Masui O. Koseki Y. Gdula M. Cerase A. (2017). PCGF3/5–PRC1 initiates Polycomb recruitment in X chromosome inactivation. Science 356, 1081–1084. 10.1126/science.aal2512 28596365 PMC6522364

[B2] An L. Dong C. Li J. Chen J. Yuan J. Huang J. (2018). RNF169 limits 53BP1 deposition at DSBs to stimulate single-strand annealing repair. Proc. Natl. Acad. Sci. U. S. A. 115, E8286–E8295. 10.1073/pnas.1804823115 30104380 PMC6126738

[B3] Anderson C. J. Baird M. R. Hsu A. Barbour E. H. Koyama Y. Borgnia M. J. (2019). Structural basis for recognition of ubiquitylated nucleosome by Dot1L methyltransferase. Cell. Rep. 26, 1681–1690. e5. 10.1016/j.celrep.2019.01.058 30759380 PMC6392056

[B4] Arrigoni R. Alam S. L. Wamstad J. A. Bardwell V. J. Sundquist W. I. Schreiber-Agus N. (2006). The Polycomb-associated protein Rybp is a ubiquitin binding protein. FEBS Lett. 580, 6233–6241. 10.1016/j.febslet.2006.10.027 17070805

[B5] Arrowsmith C. H. Schapira M. (2019). Targeting non-bromodomain chromatin readers. Nat. Struct. Mol. Biol. 26, 863–869. 10.1038/s41594-019-0290-2 31582844

[B6] Avvakumov G. V. Walker J. R. Xue S. Li Y. Duan S. Bronner C. (2008). Structural basis for recognition of hemi-methylated DNA by the SRA domain of human UHRF1. Nature 455, 822–825. 10.1038/nature07273 18772889

[B7] Batta K. Zhang Z. Yen K. Goffman D. B. Pugh B. F. (2011). Genome-wide function of H2B ubiquitylation in promoter and genic regions. Genes. Dev. 25, 2254–2265. 25/21/2254 [pii]. 10.1101/gad.177238.111 22056671 PMC3219230

[B8] Becker J. R. Clifford G. Bonnet C. Groth A. Wilson M. D. Chapman J. R. (2021). BARD1 reads H2A lysine 15 ubiquitination to direct homologous recombination. Nature 1, 433–437. –5. 10.1038/s41586-021-03776-w PMC7619424 34321663

[B9] Belotserkovskaya R. Oh S. Bondarenko V. A. Orphanides G. Studitsky V. M. Reinberg D. (2003). FACT facilitates transcription-dependent nucleosome alteration. Science 301, 1090–1093. 10.1126/science.1085703 12934006

[B10] Blackledge N. P. Farcas A. M. Kondo T. King H. W. McGouran J. F. Hanssen L. L. P. (2014). Variant PRC1 complex-dependent H2A ubiquitylation drives PRC2 recruitment and polycomb domain formation. Cell. 157, 1445–1459. 10.1016/j.cell.2014.05.004 24856970 PMC4048464

[B11] Blackledge N. P. Fursova N. A. Kelley J. R. Huseyin M. K. Feldmann A. Klose R. J. (2020). PRC1 catalytic activity is central to polycomb system function. Mol. Cell. 77, 857–874. e9. 10.1016/j.molcel.2019.12.001 31883950 PMC7033600

[B12] Blackledge N. P. Klose R. J. (2021). The molecular principles of gene regulation by Polycomb repressive complexes. Nat. Rev. Mol. Cell. Biol. 22, 815–833. 10.1038/s41580-021-00398-y 34400841 PMC7612013

[B13] Botuyan M. V. Lee J. Ward I. M. Kim J.-E. Thompson J. R. Chen J. (2006). Structural basis for the methylation state-specific recognition of histone H4-K20 by 53BP1 and Crb2 in DNA repair. Cell. 127, 1361–1373. 10.1016/j.cell.2006.10.043 17190600 PMC1804291

[B14] Campagne A. Lee M.-K. Zielinski D. Michaud A. Le Corre S. Dingli F. (2019). BAP1 complex promotes transcription by opposing PRC1-mediated H2A ubiquitylation. Nat. Commun. 10, 348. 10.1038/s41467-018-08255-x 30664650 PMC6341105

[B15] Cao Q. F. Yamamoto J. Isobe T. Tateno S. Murase Y. Chen Y. (2015). Characterization of the human transcription elongation factor Rtf1: Evidence for non-overlapping functions of Rtf1 and the Paf1 complex. Mol. Cell. Biol. 35, 3459–3470. 10.1128/MCB.00601-15 26217014 PMC4573716

[B16] Chandrasekharan M. B. Huang F. Sun Z. W. (2010). Histone H2B ubiquitination and beyond: Regulation of nucleosome stability, chromatin dynamics and the trans-histone H3 methylation. Epigenetics 5, 460–468. [pii] 10.4161/epi.5.6.12314. 10.4161/epi.5.6.12314 20523115 PMC3230548

[B17] Chandrasekharan M. B. Huang F. Sun Z. W. (2009). Ubiquitination of histone H2B regulates chromatin dynamics by enhancing nucleosome stability. Proc. Natl. Acad. Sci. U. S. A. 106, 16686–16691. 16686 [pii] 10.1073/pnas.0907862106. 10.1073/pnas.0907862106 19805358 PMC2757834

[B18] Chen H. Y. Sun J. M. Zhang Y. Davie J. R. Meistrich M. L. (1998). Ubiquitination of histone H3 in elongating spermatids of rat testes. J. Biol. Chem. 273, 13165–13169. 10.1074/jbc.273.21.13165 9582357

[B19] Cooper S. Grijzenhout A. Underwood E. Ancelin K. Zhang T. Nesterova T. B. (2016). Jarid2 binds mono-ubiquitylated H2A lysine 119 to mediate crosstalk between Polycomb complexes PRC1 and PRC2. Nat. Commun. 7, 13661. 10.1038/ncomms13661 27892467 PMC5133711

[B20] Cucinotta C. E. Hildreth A. E. McShane B. M. Shirra M. K. Arndt K. M. (2019). The nucleosome acidic patch directly interacts with subunits of the Paf1 and FACT complexes and controls chromatin architecture *in vivo* . Nucleic Acids Res. 47, 8410–8423. 10.1093/nar/gkz549 31226204 PMC6895269

[B21] Dai L. Dai Y. Han J. Huang Y. Wang L. Huang J. (2021). Structural insight into BRCA1-BARD1 complex recruitment to damaged chromatin. Mol. Cell. 81, 2765–2777. e6. 10.1016/j.molcel.2021.05.010 34102105

[B22] Dann G. P. Liszczak G. P. Bagert J. D. Muller M. M. Nguyen U. T. T. Wojcik F. (2017). ISWI chromatin remodellers sense nucleosome modifications to determine substrate preference. Nature 548, 607–611. 10.1038/nature23671 28767641 PMC5777669

[B23] de Dieuleveult M. Yen K. Hmitou I. Depaux A. Boussouar F. Dargham D. B. (2016). Genome-wide nucleosome specificity and function of chromatin remodellers in ES cells. Nature 530, 113–116. 10.1038/nature16505 26814966 PMC4871117

[B24] Debelouchina G. T. Gerecht K. Muir T. W. (2017). Ubiquitin utilizes an acidic surface patch to alter chromatin structure. Nat. Chem. Biol. 13, 105–110. 10.1038/nchembio.2235 27870837 PMC5161692

[B25] Densham R. M. Garvin A. J. Stone H. R. Strachan J. Baldock R. A. Daza-Martin M. (2016). Human BRCA1–BARD1 ubiquitin ligase activity counteracts chromatin barriers to DNA resection. Nat. Struct. Mol. Biol. 23, 647–655. 10.1038/nsmb.3236 27239795 PMC6522385

[B26] Doil C. Mailand N. Bekker-Jensen S. Menard P. Larsen D. H. Pepperkok R. (2009). RNF168 binds and amplifies ubiquitin conjugates on damaged chromosomes to allow accumulation of repair proteins. Cell. 136, 435–446. 10.1016/j.cell.2008.12.041 19203579

[B27] Edwards J. R. Yarychkivska O. Boulard M. Bestor T. H. (2017). DNA methylation and DNA methyltransferases. Epigenetics Chromatin 10, 23. 10.1186/s13072-017-0130-8 28503201 PMC5422929

[B28] Farnung L. Ochmann M. Engeholm M. Cramer P. (2021). Structural basis of nucleosome transcription mediated by Chd1 and FACT. Nat. Struct. Mol. Biol. 28, 382–387. 10.1038/s41594-021-00578-6 33846633 PMC8046669

[B29] Fierz B. Chatterjee C. McGinty R. K. Bar-Dagan M. Raleigh D. P. Muir T. W. (2011). Histone H2B ubiquitylation disrupts local and higher-order chromatin compaction. Nat. Chem. Biol. 7, 113–119. nchembio.501 [pii]. 10.1038/nchembio.501 21196936 PMC3078768

[B30] Fierz B. Kilic S. Hieb A. R. Luger K. Muir T. W. (2012). Stability of nucleosomes containing homogenously ubiquitylated H2A and H2B prepared using semisynthesis. J. Am. Chem. Soc. 134, 19548–19551. 10.1021/ja308908p 23163596 PMC3535264

[B31] Fleming A. B. Kao C. F. Hillyer C. Pikaart M. Osley M. A. (2008). H2B ubiquitylation plays a role in nucleosome dynamics during transcription elongation. Mol. Cell. 31, 57–66. S1097-2765(08)00333-X [pii]. 10.1016/j.molcel.2008.04.025 18614047

[B32] Formosa T. Winston F. (2020). The role of FACT in managing chromatin: Disruption, assembly, or repair? Nucleic Acids Res. 48, 11929–11941. 10.1093/nar/gkaa912 33104782 PMC7708052

[B33] Fradet-Turcotte A. Canny M. D. Escribano-Díaz C. Orthwein A. Leung C. C. Y. Huang H. (2013). 53BP1 is a reader of the DNA-damage-induced H2A Lys 15 ubiquitin mark. Nature 499, 50–54. 10.1038/nature12318 23760478 PMC3955401

[B34] Francis N. J. Kingston R. E. Woodcock C. L. (2004). Chromatin compaction by a polycomb group protein complex. Science 306, 1574–1577. 10.1126/science.1100576 15567868

[B35] Fuchs G. Hollander D. Voichek Y. Ast G. Oren M. (2014). Cotranscriptional histone H2B monoubiquitylation is tightly coupled with RNA polymerase II elongation rate. Genome Res. 24, 1572–1583. 10.1101/gr.176487.114 25049226 PMC4199367

[B36] Fuchs G. Oren M. (2014). Writing and reading H2B monoubiquitylation. Biochim. Biophys. Acta 1839, 694–701. 10.1016/j.bbagrm.2014.01.002 24412854

[B37] Fuchs G. Shema E. Vesterman R. Kotler E. Wolchinsky Z. Wilder S. (2012). RNF20 and USP44 regulate stem cell differentiation by modulating H2B monoubiquitylation. Mol. Cell. 46, 662–673. S1097-2765(12)00439-X [pii]. 10.1016/j.molcel.2012.05.023 22681888 PMC3374598

[B38] Fursova N. A. Turberfield A. H. Blackledge N. P. Findlater E. L. Lastuvkova A. Huseyin M. K. (2021). BAP1 constrains pervasive H2AK119ub1 to control the transcriptional potential of the genome. Genes. Dev. 35, 749–770. 10.1101/gad.347005.120 33888563 PMC8091973

[B39] Fyodorov D. V. Zhou B.-R. Skoultchi A. I. Bai Y. (2018). Emerging roles of linker histones in regulating chromatin structure and function. Nat. Rev. Mol. Cell. Biol. 19, 192–206. 10.1038/nrm.2017.94 29018282 PMC5897046

[B40] Goldknopf I. L. Busch H. (1977). Isopeptide linkage between nonhistone and histone 2A polypeptides of chromosomal conjugate-protein A24. Proc. Natl. Acad. Sci. U. S. A. 74, 864–868. 10.1073/pnas.74.3.864 265581 PMC430507

[B41] Grau D. J. Chapman B. A. Garlick J. D. Borowsky M. Francis N. J. Kingston R. E. (2011). Compaction of chromatin by diverse Polycomb group proteins requires localized regions of high charge. Genes. Dev. 25, 2210–2221. 10.1101/gad.17288211 22012622 PMC3205590

[B42] Hauri S. Comoglio F. Seimiya M. Gerstung M. Glatter T. Hansen K. (2016). A high-density map for navigating the human polycomb complexome. Cell. Rep. 17, 583–595. 10.1016/j.celrep.2016.08.096 27705803

[B43] Helchowski C. M. Skow L. F. Roberts K. H. Chute C. L. Canman C. E. (2013). A small ubiquitin binding domain inhibits ubiquitin-dependent protein recruitment to DNA repair foci. Cell. Cycle 12, 3749–3758. 10.4161/cc.26640 24107634 PMC3905067

[B44] Hennig B. P. Bendrin K. Zhou Y. Fischer T. (2012). Chd1 chromatin remodelers maintain nucleosome organization and repress cryptic transcription. EMBO Rep. 13, 997–1003. embor2012146 [pii]. 10.1038/embor.2012.146 23032292 PMC3492713

[B45] Hsu P. L. Shi H. Leonen C. Kang J. Chatterjee C. Zheng N. (2019). Structural basis of H2B ubiquitination-dependent H3K4 methylation by COMPASS. Mol. Cell. 76, 712–723. e4. 10.1016/j.molcel.2019.10.013 31733991 PMC6948180

[B46] Hu Q. Botuyan M. V. Cui G. Zhao D. Mer G. (2017). Mechanisms of ubiquitin-nucleosome recognition and regulation of 53BP1 chromatin recruitment by rnf168/169 and RAD18. Mol. Cell. 66, 473–487. e9. 10.1016/j.molcel.2017.04.009 28506460 PMC5523955

[B47] Hu Q. Botuyan M. V. Zhao D. Cui G. Mer E. Mer G. (2021). Mechanisms of BRCA1–BARD1 nucleosome recognition and ubiquitylation. Nature 596, 438–443. 10.1038/s41586-021-03716-8 34321665 PMC8680157

[B48] Huang J. Huen M. S. Y. Kim H. Leung C. C. Y. Glover J. N. M. Yu X. (2009). RAD18 transmits DNA damage signalling to elicit homologous recombination repair. Nat. Cell. Biol. 11, 592–603. 10.1038/ncb1865 19396164 PMC2743127

[B49] Husnjak K. Dikic I. (2012). Ubiquitin-binding proteins: Decoders of ubiquitin-mediated cellular functions. Annu. Rev. Biochem. 81, 291–322. 10.1146/annurev-biochem-051810-094654 22482907

[B50] Hwang W. W. Venkatasubrahmanyam S. Ianculescu A. G. Tong A. Boone C. Madhani H. D. (2003). A conserved RING finger protein required for histone H2B monoubiquitination and cell size control. Mol. Cell. 11, 261–266. 10.1016/s1097-2765(02)00826-2 12535538

[B51] Ishiyama S. Nishiyama A. Saeki Y. Moritsugu K. Morimoto D. Yamaguchi L. (2017). Structure of the Dnmt1 reader module complexed with a unique two-mono-ubiquitin mark on histone H3 reveals the basis for DNA methylation maintenance. Mol. Cell. 68, 350–360. e7. 10.1016/j.molcel.2017.09.037 29053958

[B52] Isono K. Endo T. A. Ku M. Yamada D. Suzuki R. Sharif J. (2013). SAM domain polymerization links subnuclear clustering of PRC1 to gene silencing. Dev. Cell. 26, 565–577. 10.1016/j.devcel.2013.08.016 24091011

[B53] Jang S. Kang C. Yang H.-S. Jung T. Hebert H. Chung K. Y. (2019). Structural basis of recognition and destabilization of the histone H2B ubiquitinated nucleosome by the DOT1L histone H3 Lys79 methyltransferase. Genes. Dev. 33, 620–625. 10.1101/gad.323790.118 30923167 PMC6546062

[B54] Jeronimo C. Angel A. Nguyen V. Q. Kim J. M. Poitras C. Lambert E. (2021). FACT is recruited to the +1 nucleosome of transcribed genes and spreads in a Chd1-dependent manner. Mol. Cell. 81, 3542–3559.e11. 10.1016/j.molcel.2021.07.010 34380014 PMC9149603

[B55] Kalb R. Latwiel S. Baymaz H. I. Jansen P. W. T. C. Müller C. W. Vermeulen M. (2014a). Histone H2A monoubiquitination promotes histone H3 methylation in Polycomb repression. Nat. Struct. Mol. Biol. 21, 569–571. 10.1038/nsmb.2833 24837194

[B56] Kalb R. Mallery D. L. Larkin C. Huang J. T. J. Hiom K. (2014b). BRCA1 is a histone-H2A-specific ubiquitin ligase. Cell. Rep. 8, 999–1005. 10.1016/j.celrep.2014.07.025 25131202 PMC4382519

[B57] Karpiuk O. Najafova Z. Kramer F. Hennion M. Galonska C. Konig A. (2012). The histone H2B monoubiquitination regulatory pathway is required for differentiation of multipotent stem cells. Mol. Cell. 46, 705–713. S1097-2765(12)00438-8 [pii]. 10.1016/j.molcel.2012.05.022 22681891

[B58] Kasinath V. Beck C. Sauer P. Poepsel S. Kosmatka J. Faini M. (2021). JARID2 and AEBP2 regulate PRC2 in the presence of H2AK119ub1 and other histone modifications. Science 371, eabc3393. 10.1126/science.abc3393 33479123 PMC7993630

[B59] Kemble D. J. McCullough L. L. Whitby F. G. Formosa T. Hill C. P. (2015). FACT disrupts nucleosome structure by binding H2A-H2B with conserved peptide motifs. Mol. Cell. 60, 294–306. 10.1016/j.molcel.2015.09.008 26455391 PMC4620744

[B60] Kim J. Kim J. A. McGinty R. K. Nguyen U. T. Muir T. W. Allis C. D. (2013). The n-SET domain of Set1 regulates H2B ubiquitylation-dependent H3K4 methylation. Mol. Cell. 49, 1121–1133. S1097-2765(13)00098-1 [pii]. 10.1016/j.molcel.2013.01.034 23453808 PMC3615140

[B61] Kitevski-LeBlanc J. Fradet-Turcotte A. Kukic P. Wilson M. D. Portella G. Yuwen T. (2017). The RNF168 paralog RNF169 defines a new class of ubiquitylated histone reader involved in the response to DNA damage. eLife 6, e23872. 10.7554/eLife.23872 28406400 PMC5426901

[B62] Komander D. Rape M. (2012). The ubiquitin code. Annu. Rev. Biochem. 81, 203–229. 10.1146/annurev-biochem-060310-170328 22524316

[B63] Kornberg R. D. Lorch Y. (2020). Primary role of the nucleosome. Mol. Cell. 79, 371–375. 10.1016/j.molcel.2020.07.020 32763226

[B64] Krajewski W. A. Li J. Dou Y. (2018). Effects of histone H2B ubiquitylation on the nucleosome structure and dynamics. Nucleic Acids Res. 46, 7631–7642. 10.1093/nar/gky526 29931239 PMC6125632

[B65] Krivtsov A. V. Hoshii T. Armstrong S. A. (2017). Mixed-lineage leukemia fusions and chromatin in leukemia. Cold Spring Harb. Perspect. Med. 7, a026658. 10.1101/cshperspect.a026658 28242784 PMC5666623

[B66] Kwon M. Park K. Hyun K. Lee J. H. Zhou L. Cho Y. W. (2020). H2B ubiquitylation enhances H3K4 methylation activities of human KMT2 family complexes. Nucleic Acids Res. 48, 5442–5456. 10.1093/nar/gkaa317 32365172 PMC7261165

[B67] Larson A. G. Elnatan D. Keenen M. M. Trnka M. J. Johnston J. B. Burlingame A. L. (2017). Liquid droplet formation by HP1α suggests a role for phase separation in heterochromatin. Nature 547, 236–240. 10.1038/nature22822 28636604 PMC5606208

[B68] Lee J. S. Garrett A. S. Yen K. Takahashi Y. H. Hu D. Jackson J. (2012). Codependency of H2B monoubiquitination and nucleosome reassembly on Chd1. Genes. Dev. 26, 914–919. 10.1101/gad.186841.112 22549955 PMC3347789

[B69] Leonhardt H. Page A. W. Weier H.-U. Bestor T. H. (1992). A targeting sequence directs DNA methyltransferase to sites of DNA replication in mammalian nuclei. Cell. 71, 865–873. 10.1016/0092-8674(92)90561-P 1423634

[B70] Levendosky R. F. Sabantsev A. Deindl S. Bowman G. D. (2016). The Chd1 chromatin remodeler shifts hexasomes unidirectionally. Elife 5, e21356. 10.7554/eLife.21356 28032848 PMC5226652

[B71] Li T. Wang L. Du Y. Xie S. Yang X. Lian F. (2018). Structural and mechanistic insights into UHRF1-mediated DNMT1 activation in the maintenance DNA methylation. Nucleic Acids Res. 46, 3218–3231. 10.1093/nar/gky104 29471350 PMC5887372

[B72] Liu Y. Zhou K. Zhang N. Wei H. Tan Y. Z. Zhang Z. (2020). FACT caught in the act of manipulating the nucleosome. Nature 577, 426–431. 10.1038/s41586-019-1820-0 31775157 PMC7441595

[B73] Marsh D. J. Dickson K. A. (2019). Writing histone monoubiquitination in human malignancy-the role of RING finger E3 ubiquitin ligases. Genes. (Basel) 10, E67. 10.3390/genes10010067 30669413 PMC6356280

[B74] Mashtalir N. Dao H. T. Sankar A. Liu H. Corin A. J. Bagert J. D. (2021). Chromatin landscape signals differentially dictate the activities of mSWI/SNF family complexes. Science 373, 306–315. 10.1126/science.abf8705 34437148 PMC8390793

[B75] Mattiroli F. Penengo L. (2021). Histone ubiquitination: An integrative signaling platform in genome stability. Trends Genet. 37, 566–581. 10.1016/j.tig.2020.12.005 33485674

[B76] Mattiroli F. Vissers J. H. A. van Dijk W. J. Ikpa P. Citterio E. Vermeulen W. (2012). RNF168 ubiquitinates K13-15 on H2A/H2AX to drive DNA damage signaling. Cell. 150, 1182–1195. 10.1016/j.cell.2012.08.005 22980979

[B77] Mbogning J. Nagy S. Page V. Schwer B. Shuman S. Fisher R. P. (2013). The PAF complex and Prf1/Rtf1 delineate distinct Cdk9-dependent pathways regulating transcription elongation in fission yeast. PLoS Genet. 9, e1004029. 10.1371/journal.pgen.1004029 24385927 PMC3873232

[B78] McGinty R. K. Kim J. Chatterjee C. Roeder R. G. Muir T. W. (2008). Chemically ubiquitylated histone H2B stimulates hDot1L-mediated intranucleosomal methylation. Nature 453, 812–816. nature06906 [pii]. 10.1038/nature06906 18449190 PMC3774535

[B79] McGinty R. K. Tan S. (2021). Principles of nucleosome recognition by chromatin factors and enzymes. Curr. Opin. Struct. Biol. 71, 16–26. 10.1016/j.sbi.2021.05.006 34198054 PMC8648869

[B80] Mevissen T. E. T. Komander D. (2017). Mechanisms of deubiquitinase specificity and regulation. Annu. Rev. Biochem. 86, 159–192. 10.1146/annurev-biochem-061516-044916 28498721

[B81] Minsky N. Shema E. Field Y. Schuster M. Segal E. Oren M. (2008). Monoubiquitinated H2B is associated with the transcribed region of highly expressed genes in human cells. Nat. Cell. Biol. 10, 483–488. ncb1712 [pii] 10.1038/ncb1712. 10.1038/ncb1712 18344985

[B82] Mohn F. Weber M. Rebhan M. Roloff T. C. Richter J. Stadler M. B. (2008). Lineage-specific polycomb targets and de novo DNA methylation define restriction and potential of neuronal progenitors. Mol. Cell. 30, 755–766. 10.1016/j.molcel.2008.05.007 18514006

[B83] Morgan M. T. Haj-Yahya M. Ringel A. E. Bandi P. Brik A. Wolberger C. (2016). Structural basis for histone H2B deubiquitination by the SAGA DUB module. Science 351, 725–728. 10.1126/science.aac5681 26912860 PMC4863942

[B84] Morrison E. A. Sanchez J. C. Ronan J. L. Farrell D. P. Varzavand K. Johnson J. K. (2017). DNA binding drives the association of BRG1/hBRM bromodomains with nucleosomes. Nat. Commun. 8, 16080. 10.1038/ncomms16080 28706277 PMC5519978

[B85] Murawska M. Schauer T. Matsuda A. Wilson M. D. Pysik T. Wojcik F. (2020). The chaperone FACT and histone H2B ubiquitination maintain *S. pombe* genome architecture through genic and subtelomeric functions. Mol. Cell. 77, 501–513. e7. 10.1016/j.molcel.2019.11.016 31837996 PMC7007867

[B86] Musselman C. A. Khorasanizadeh S. Kutateladze T. G. (2014). Towards understanding methyllysine readout. Biochim. Biophys. Acta 1839, 686–693. 10.1016/j.bbagrm.2014.04.001 24727128 PMC4453862

[B87] Musselman C. A. Lalonde M.-E. Côté J. Kutateladze T. G. (2012). Perceiving the epigenetic landscape through histone readers. Nat. Struct. Mol. Biol. 19, 1218–1227. 10.1038/nsmb.2436 23211769 PMC3645987

[B88] Nakamura K. Saredi G. Becker J. R. Foster B. M. Nguyen N. V. Beyer T. E. (2019). H4K20me0 recognition by BRCA1–BARD1 directs homologous recombination to sister chromatids. Nat. Cell. Biol. 21, 311–318. 10.1038/s41556-019-0282-9 30804502 PMC6420097

[B89] Nambiar T. S. Billon P. Diedenhofen G. Hayward S. B. Taglialatela A. Cai K. (2019). Stimulation of CRISPR-mediated homology-directed repair by an engineered RAD18 variant. Nat. Commun. 10, 3395. 10.1038/s41467-019-11105-z 31363085 PMC6667477

[B90] Nickel B. E. Allis C. D. Davie J. R. (1989). Ubiquitinated histone H2B is preferentially located in transcriptionally active chromatin. Biochemistry 28, 958–963. 10.1021/bi00429a006 2713375

[B91] Nishiyama A. Yamaguchi L. Nakanishi M. (2016). Regulation of maintenance DNA methylation via histone ubiquitylation. J. Biochem. 159, 9–15. 10.1093/jb/mvv113 26590302 PMC4882649

[B92] Nishiyama A. Yamaguchi L. Sharif J. Johmura Y. Kawamura T. Nakanishi K. (2013). Uhrf1-dependent H3K23 ubiquitylation couples maintenance DNA methylation and replication. Nature 502, 249–253. 10.1038/nature12488 24013172

[B93] Nune M. Morgan M. T. Connell Z. McCullough L. Jbara M. Sun H. (2019). FACT and Ubp10 collaborate to modulate H2B deubiquitination and nucleosome dynamics. Elife 8, e40988. 10.7554/eLife.40988 30681413 PMC6372288

[B94] Oya E. Nakagawa R. Yoshimura Y. Tanaka M. Nishibuchi G. Machida S. (2019). H3K14 ubiquitylation promotes H3K9 methylation for heterochromatin assembly. EMBO Rep. 20, e48111. 10.15252/embr.201948111 31468675 PMC6776926

[B95] Panier S. Boulton S. J. (2014). Double-strand break repair: 53BP1 comes into focus. Nat. Rev. Mol. Cell. Biol. 15, 7–18. 10.1038/nrm3719 24326623

[B96] Panier S. Ichijima Y. Fradet-Turcotte A. Leung C. C. Y. Kaustov L. Arrowsmith C. H. (2012). Tandem protein interaction modules organize the ubiquitin-dependent response to DNA double-strand breaks. Mol. Cell. 47, 383–395. 10.1016/j.molcel.2012.05.045 22742833

[B97] Pavri R. Zhu B. Li G. Trojer P. Mandal S. Shilatifard A. (2006). Histone H2B monoubiquitination functions cooperatively with FACT to regulate elongation by RNA polymerase II. Cell. 125, 703–717. 10.1016/j.cell.2006.04.029 16713563

[B98] Pellegrino S. Michelena J. Teloni F. Imhof R. Altmeyer M. (2017). Replication-coupled dilution of H4K20me2 guides 53BP1 to pre-replicative chromatin. Cell. Rep. 19, 1819–1831. 10.1016/j.celrep.2017.05.016 28564601 PMC5857200

[B99] Plys A. J. Davis C. P. Kim J. Rizki G. Keenen M. M. Marr S. K. (2019). Phase separation of Polycomb-repressive complex 1 is governed by a charged disordered region of CBX2. Genes. Dev. 33, 799–813. 10.1101/gad.326488.119 31171700 PMC6601514

[B100] Pointner J. Persson J. Prasad P. Norman-Axelsson U. Stralfors A. Khorosjutina O. (2012). CHD1 remodelers regulate nucleosome spacing *in vitro* and align nucleosomal arrays over gene coding regions in *S. pombe* . Embo J. 31, 4388–4403. 10.1038/emboj.2012.289 23103765 PMC3512388

[B101] Poulsen M. Lukas C. Lukas J. Bekker-Jensen S. Mailand N. (2012). Human RNF169 is a negative regulator of the ubiquitin-dependent response to DNA double-strand breaks. J. Cell. Biol. 197, 189–199. 10.1083/jcb.201109100 22492721 PMC3328375

[B102] Qin W. Wolf P. Liu N. Link S. Smets M. Mastra F. L. (2015). DNA methylation requires a DNMT1 ubiquitin interacting motif (UIM) and histone ubiquitination. Cell. Res. 25, 911–929. 10.1038/cr.2015.72 26065575 PMC4528052

[B103] Ramachandran S. Ahmad K. Henikoff S. (2017). Transcription and remodeling produce asymmetrically unwrapped nucleosomal intermediates. Mol. Cell. 68, 1038–1053. e4. 10.1016/j.molcel.2017.11.015 29225036 PMC6421108

[B104] Richly H. Rocha-Viegas L. Ribeiro J. D. Demajo S. Gundem G. Lopez-Bigas N. (2010). Transcriptional activation of polycomb-repressed genes by ZRF1. Nature 468, 1124–1128. 10.1038/nature09574 21179169

[B105] Robzyk K. Recht J. Osley M. A. (2000). Rad6-dependent ubiquitination of histone H2B in yeast. Science 287, 501–504. 10.1126/science.287.5452.501 10642555

[B106] Rothbart S. B. Dickson B. M. Ong M. S. Krajewski K. Houliston S. Kireev D. B. (2013). Multivalent histone engagement by the linked tandem Tudor and PHD domains of UHRF1 is required for the epigenetic inheritance of DNA methylation. Genes. Dev. 27, 1288–1298. 10.1101/gad.220467.113 23752590 PMC3690401

[B107] Rothbart S. B. Krajewski K. Nady N. Tempel W. Xue S. Badeaux A. I. (2012). Association of UHRF1 with methylated H3K9 directs the maintenance of DNA methylation. Nat. Struct. Mol. Biol. 19, 1155–1160. 10.1038/nsmb.2391 23022729 PMC3492551

[B108] Rothbart S. B. Strahl B. D. (2014). Interpreting the language of histone and DNA modifications. Biochim. Biophys. Acta 1839, 627–643. 10.1016/j.bbagrm.2014.03.001 24631868 PMC4099259

[B109] Salas-Lloret D. González-Prieto R. (2022). Insights in post-translational modifications: Ubiquitin and SUMO. Int. J. Mol. Sci. 23, 3281. 10.3390/ijms23063281 35328702 PMC8952880

[B110] Sanso M. Lee K. M. Viladevall L. Jacques P. E. Page V. Nagy S. (2012). A positive feedback loop links opposing functions of P-TEFb/Cdk9 and histone H2B ubiquitylation to regulate transcript elongation in fission yeast. PLoS Genet. 8, e1002822. [pii]. 10.1371/journal.pgen.1002822 22876190 PMC3410854

[B111] Sanso M. Parua P. K. Pinto D. Svensson J. P. Page V. Bitton D. A. (2020). Cdk9 and H2Bub1 signal to Clr6-CII/Rpd3S to suppress aberrant antisense transcription. Nucleic Acids Res. 48, 7154–7168. 10.1093/nar/gkaa474 32496538 PMC7367204

[B112] Sanulli S. Trnka M. J. Dharmarajan V. Tibble R. W. Pascal B. D. Burlingame A. L. (2019). HP1 reshapes nucleosome core to promote phase separation of heterochromatin. Nature 575, 390–394. 10.1038/s41586-019-1669-2 31618757 PMC7039410

[B113] Saredi G. Huang H. Hammond C. M. Alabert C. Bekker-Jensen S. Forne I. (2016). H4K20me0 marks post-replicative chromatin and recruits the TONSL–MMS22L DNA repair complex. Nature 534, 714–718. 10.1038/nature18312 27338793 PMC4939875

[B114] Shema-Yaacoby E. Nikolov M. Haj-Yahya M. Siman P. Allemand E. Yamaguchi Y. (2013). Systematic identification of proteins binding to chromatin-embedded ubiquitylated H2B reveals recruitment of SWI/SNF to regulate transcription. Cell. Rep. 4, 601–608. S2211-1247(13)00361-6 [pii]. 10.1016/j.celrep.2013.07.014 23933260

[B115] Shilatifard A. (2012). The COMPASS family of histone H3K4 methylases: Mechanisms of regulation in development and disease pathogenesis. Annu. Rev. Biochem. 81, 65–95. 10.1146/annurev-biochem-051710-134100 22663077 PMC4010150

[B116] Skrajna A. Goldfarb D. Kedziora K. M. Cousins E. M. Grant G. D. Spangler C. J. (2020). Comprehensive nucleosome interactome screen establishes fundamental principles of nucleosome binding. Nucleic Acids Res. 48, 9415–9432. 10.1093/nar/gkaa544 32658293 PMC7515726

[B117] Smolle M. Venkatesh S. Gogol M. M. Li H. Zhang Y. Florens L. (2012). Chromatin remodelers Isw1 and Chd1 maintain chromatin structure during transcription by preventing histone exchange. Nat. Struct. Mol. Biol. 19, 884–892. nsmb.2312 [pii] 10.1038/nsmb.2312. 10.1038/nsmb.2312 22922743 PMC3560298

[B118] Spencer D. H. Russler-Germain D. A. Ketkar S. Helton N. M. Lamprecht T. L. Fulton R. S. (2017). CpG island hypermethylation mediated by DNMT3A is a consequence of AML progression. Cell. 168, 801–816. e13. 10.1016/j.cell.2017.01.021 28215704 PMC5328582

[B119] Stewart G. S. Panier S. Townsend K. Al-Hakim A. K. Kolas N. K. Miller E. S. (2009). The RIDDLE syndrome protein mediates a ubiquitin-dependent signaling cascade at sites of DNA damage. Cell. 136, 420–434. 10.1016/j.cell.2008.12.042 19203578

[B120] Stirpe A. Guidotti N. Northall S. J. Kilic S. Hainard A. Vadas O. (2021). SUV39 SET domains mediate crosstalk of heterochromatic histone marks. eLife 10, e62682. 10.7554/eLife.62682 34524082 PMC8443253

[B121] Strahl B. D. Allis C. D. (2000). The language of covalent histone modifications. Nature 403, 41–45. 10.1038/47412 10638745

[B122] Strom A. R. Emelyanov A. V. Mir M. Fyodorov D. V. Darzacq X. Karpen G. H. (2017). Phase separation drives heterochromatin domain formation. Nature 547, 241–245. 10.1038/nature22989 28636597 PMC6022742

[B123] Sundaramoorthy R. Hughes A. L. El-Mkami H. Norman D. G. Ferreira H. Owen-Hughes T. (2018). Structure of the chromatin remodelling enzyme Chd1 bound to a ubiquitinylated nucleosome. eLife 7, e35720. 10.7554/eLife.35720 30079888 PMC6118821

[B124] Tamburri S. Lavarone E. Fernández-Pérez D. Conway E. Zanotti M. Manganaro D. (2020). Histone H2AK119 mono-ubiquitination is essential for polycomb-mediated transcriptional repression. Mol. Cell. 77, 840–856. e5. 10.1016/j.molcel.2019.11.021 31883952 PMC7033561

[B125] Tanny J. C. (2014). Chromatin modification by the RNA Polymerase II elongation complex. Transcription 5, e988093. 10.4161/21541264.2014.988093 25494544 PMC4581355

[B126] Tanny J. C. Erdjument-Bromage H. Tempst P. Allis C. D. (2007). Ubiquitylation of histone H2B controls RNA polymerase II transcription elongation independently of histone H3 methylation. Genes. Dev. 21, 835–847. gad.1516207 [pii] 10.1101/gad.1516207. 10.1101/gad.1516207 17374714 PMC1838534

[B127] Tarcic O. Granit R. Z. Pateras I. S. Masury H. Maly B. Zwang Y. (2017). RNF20 and histone H2B ubiquitylation exert opposing effects in Basal-Like versus luminal breast cancer. Cell. Death Differ. 24, 694–704. 10.1038/cdd.2016.126 28157208 PMC5384017

[B128] Tarcic O. Pateras I. S. Cooks T. Shema E. Kanterman J. Ashkenazi H. (2016). RNF20 links histone H2B ubiquitylation with inflammation and inflammation-associated cancer. Cell. Rep. 14, 1462–1476. 10.1016/j.celrep.2016.01.020 26854224 PMC4761112

[B129] Tavares L. Dimitrova E. Oxley D. Webster J. Poot R. Demmers J. (2012). RYBP-PRC1 complexes mediate H2A ubiquitylation at polycomb target sites independently of PRC2 and H3K27me3. Cell. 148, 664–678. 10.1016/j.cell.2011.12.029 22325148 PMC3281992

[B130] Uckelmann M. Densham R. M. Baas R. Winterwerp H. H. K. Fish A. Sixma T. K. (2018). USP48 restrains resection by site-specific cleavage of the BRCA1 ubiquitin mark from H2A. Nat. Commun. 9, 229. 10.1038/s41467-017-02653-3 29335415 PMC5768779

[B131] Valencia-Sánchez M. I. De Ioannes P. Wang M. Vasilyev N. Chen R. Nudler E. (2019). Structural basis of Dot1L stimulation by histone H2B lysine 120 ubiquitination. Mol. Cell. 74, 1010–1019. e6. 10.1016/j.molcel.2019.03.029 30981630 PMC7009778

[B132] Van Oss S. B. Shirra M. K. Bataille A. R. Wier A. D. Yen K. Vinayachandran V. (2016). The histone modification domain of Paf1 complex subunit Rtf1 directly stimulates H2B ubiquitylation through an interaction with Rad6. Mol. Cell. 64, 815–825. 10.1016/j.molcel.2016.10.008 27840029 PMC5131541

[B133] Vaughan R. M. Kupai A. Rothbart S. B. (2021). Chromatin regulation through ubiquitin and ubiquitin-like histone modifications. Trends biochem. Sci. 46, 258–269. 10.1016/j.tibs.2020.11.005 33308996 PMC7954875

[B134] Vlaming H. van Leeuwen F. (2016). The upstreams and downstreams of H3K79 methylation by DOT1L. Chromosoma 125, 593–605. 10.1007/s00412-015-0570-5 26728620

[B135] Wang H. Wang L. Erdjument-Bromage H. Vidal M. Tempst P. Jones R. S. (2004). Role of histone H2A ubiquitination in Polycomb silencing. Nature 431, 873–878. 10.1038/nature02985 15386022

[B136] Weinberg D. N. Rosenbaum P. Chen X. Barrows D. Horth C. Marunde M. R. (2021). Two competing mechanisms of DNMT3A recruitment regulate the dynamics of de novo DNA methylation at PRC1-targeted CpG islands. Nat. Genet. 53, 794–800. 10.1038/s41588-021-00856-5 33986537 PMC8283687

[B137] Wilson M. D. Benlekbir S. Fradet-Turcotte A. Sherker A. Julien J.-P. McEwan A. (2016). The structural basis of modified nucleosome recognition by 53BP1. Nature 536, 100–103. 10.1038/nature18951 27462807

[B138] Witus S. R. Stewart M. D. Klevit R. E. (2021). The BRCA1/BARD1 ubiquitin ligase and its substrates. Biochem. J. 478, 3467–3483. 10.1042/BCJ20200864 34591954 PMC8763022

[B139] Worden E. J. Hoffmann N. A. Hicks C. W. Wolberger C. (2019). Mechanism of cross-talk between H2B ubiquitination and H3 methylation by Dot1L. Cell. 176, 1490–1501. 10.1016/j.cell.2019.02.002 30765112 PMC6498860

[B140] Worden E. J. Wolberger C. (2019). Activation and regulation of H2B-Ubiquitin-dependent histone methyltransferases. Curr. Opin. Struct. Biol. 59, 98–106. 10.1016/j.sbi.2019.05.009 31229920 PMC6888998

[B141] Worden E. J. Zhang X. Wolberger C. (2020). Structural basis for COMPASS recognition of an H2B-ubiquitinated nucleosome. Elife 9, e53199. 10.7554/eLife.53199 31922488 PMC7039682

[B142] Wu L. Lee S. Y. Zhou B. Nguyen U. T. Muir T. W. Tan S. (2013). ASH2L regulates ubiquitylation signaling to MLL: trans-regulation of H3 K4 methylation in higher eukaryotes. Mol. Cell. 49, 1108–1120. S1097-2765(13)00097-X [pii]. 10.1016/j.molcel.2013.01.033 23453805 PMC3615107

[B143] Xiao X. Liu C. Pei Y. Wang Y.-Z. Kong J. Lu K. (2020). Histone H2A ubiquitination reinforces mechanical stability and asymmetry at the single-nucleosome level. J. Am. Chem. Soc. 142, 3340–3345. 10.1021/jacs.9b12448 32003988

[B144] Xue H. Yao T. Cao M. Zhu G. Li Y. Yuan G. (2019). Structural basis of nucleosome recognition and modification by MLL methyltransferases. Nature 573, 445–449. 10.1038/s41586-019-1528-1 31485071

[B145] Yaakov G. Jonas F. Barkai N. (2021). Measurement of histone replacement dynamics with genetically encoded exchange timers in yeast. Nat. Biotechnol. 39, 1434–1443. 10.1038/s41587-021-00959-8 34239087

[B146] Zhang X. Li B. Rezaeian A. H. Xu X. Chou P.-C. Jin G. (2017). H3 ubiquitination by NEDD4 regulates H3 acetylation and tumorigenesis. Nat. Commun. 8, 14799. 10.1038/ncomms14799 28300060 PMC5357315

[B147] Zhang Z. Jones A. E. Wu W. Kim J. Kang Y. Bi X. (2017). Role of remodeling and spacing factor 1 in histone H2A ubiquitination-mediated gene silencing. Proc. Natl. Acad. Sci. U. S. A. 114, E7949-E7958–E7958. 10.1073/pnas.1711158114 28855339 PMC5617306

[B148] Zhao J. Wang M. Chang L. Yu J. Song A. Liu C. (2020). RYBP/YAF2-PRC1 complexes and histone H1-dependent chromatin compaction mediate propagation of H2AK119ub1 during cell division. Nat. Cell. Biol. 22, 439–452. 10.1038/s41556-020-0484-1 32203418

[B149] Zhou L. Holt M. T. Ohashi N. Zhao A. Müller M. M. Wang B. (2016). Evidence that ubiquitylated H2B corrals hDot1L on the nucleosomal surface to induce H3K79 methylation. Nat. Commun. 7, 10589. 10.1038/ncomms10589 26830124 PMC4740876

[B150] Zhu Q. Pao G. M. Huynh A. M. Suh H. Tonnu N. Nederlof P. M. (2011). BRCA1 tumour suppression occurs via heterochromatin-mediated silencing. Nature 477, 179–184. 10.1038/nature10371 21901007 PMC3240576

